# The Structure–Decoding–Conversion–Effect Paradigm of Natural Polysaccharides for Gut Microbiota Remodeling in Ulcerative Colitis

**DOI:** 10.3390/nu18081297

**Published:** 2026-04-20

**Authors:** Xin-Qian Rong, Xiao-Meng Zhang, Lan Yan, Yong Tan, Cheng Lu

**Affiliations:** Institute of Basic Research in Clinical Medicine, China Academy of Chinese Medical Sciences, Beijing 100700, China; rongxinqian2016@163.com (X.-Q.R.); zhxmzoe@163.com (X.-M.Z.); yanlan1998@163.com (L.Y.)

**Keywords:** ulcerative colitis, natural polysaccharides, gut microbiota, structure–activity relationship, prebiotics

## Abstract

Ulcerative colitis (UC), a chronic inflammatory bowel disease, is closely associated with disturbances in the gut microbiota. Natural polysaccharides, owing to their unique “indigestibility” and prebiotic properties, represent a potential strategy for intervening in UC by remodeling the gut microecology. This review summarizes the mechanisms by which natural polysaccharides alleviate UC through modulation of the gut microbiota, with a particular focus on the structure–activity relationship between the structural features of natural polysaccharides and their microbiota-regulating functions. Analytical studies indicate that polysaccharides with distinct structures can be recognized and degraded by specific carbohydrate-active enzymes (CAZymes) in the gut microorganisms, leading to the targeted enrichment of beneficial genera such as *Roseburia*, *Lactobacillus*, and *Akkermansia*, while simultaneously suppressing pro-inflammatory genera such as *Escherichia*–*Shigella* and *Helicobacter*. This structure-dependent microbial remodeling ultimately enhances the production of key metabolites and exerts comprehensive therapeutic effects, including repair of the intestinal barrier, suppression of excessive inflammation, and alleviation of oxidative stress, via activation of signaling pathways such as AMP-activated protein kinase (AMPK) and nuclear factor erythroid 2-related factor 2 (Nrf2) and inhibition of pathways such as nuclear factor kappa-B (NF-κB). By exploring the paradigm of “Structure–Decoding–Conversion–Effect” based on precise microecological regulation of polysaccharide structures, this paper provides a crucial theoretical foundation and design strategy for developing targeted microecological interventions.

## 1. Introduction

Ulcerative colitis (UC), a primary clinical subtype of inflammatory bowel disease, is characterized by continuous inflammation, diffuse damage, and ulceration of the colonic mucosa, with persistent or recurrent diarrhea, abdominal pain, and bloody stools as its core clinical symptoms [1]. As of 2023, the global prevalence of UC is estimated to exceed 5 million cases, with incidence rates continuing to rise, posing a growing health challenge and economic burden on public health systems [2]. The precise etiology and pathogenesis of UC remain incompletely understood, but are widely considered to result from the complex interplay of genetic susceptibility, environmental factors, gut microbiota dysbiosis, and abnormal mucosal immune responses [3]. Among these, disruption of gut microbial community homeostasis is recognized as a pivotal driver in the onset and progression of UC [4]. Microbiome profiling studies of clinical patients have consistently demonstrated that the gut microbiota of UC patients typically exhibits a depletion of commensal bacteria and an increase in opportunistic pathogens, shifting from a symbiotic, fermentation-dominated ecology to one dominated by inflammation-adapted opportunists [5]. Studies have shown that patients with UC exhibit reduced abundances of gut SCFA-producing bacteria (e.g., *Prevotella*, *Coprococcus*, *Faecalibacterium*, and *Roseburia*), an expansion of Proteobacteria, increased levels of opportunistic pathogens such as *Escherichia*–*Shigella*, and decreased community diversity and functional redundancy [6,7]. This ecological imbalance can trigger aberrant inflammatory responses and dysregulated immune activation, further inducing intestinal mucosal damage and inflammation [8]; moreover, restoring microbial balance via intestinal microbiota transplantation correspondingly attenuates cytokine levels and improves mucosal barrier function in UC [9]. However, current therapeutic regimens largely focus on symptom control [10], while systematic strategies for restructuring the functional ecological niches of the gut microbiota warrant further in-depth investigation [11].

In recent years, natural polysaccharides have emerged as a prominent research focus within the realm of microecological intervention strategies for UC, owing to their notable microbiota-modulating and anti-inflammatory activities [12]. Compared to direct administration of probiotics or fecal microbiota transplantation, polysaccharide-based interventions do not introduce live organisms but rather reshape microbial interaction networks via a carbon-source strategy, offering greater stability and safety [13]. Existing research has confirmed that natural polysaccharides can restore gut microecological balance in the context of UC by increasing the abundance of probiotics/commensals, inhibiting the proliferation of potential pathogens, and promoting the production of short-chain fatty acids (SCFAs) [14]. The underlying mechanism hinges on the human body’s lack of enzymatic systems to degrade non-starch polysaccharides, allowing the majority of these macromolecules to reach the colon intact, where they are selectively utilized by gut microbes, thereby modulating microbial structure and function [15]. Furthermore, certain polysaccharides with specific structures or their degradation fragments can directly interact with pattern recognition receptors on immune cells, subsequently regulating the host’s immune response [16]. Due to the core characteristic of their “indigestibility” for humans, natural polysaccharides function as potential prebiotics that ameliorate disease by modulating the composition and metabolism of the gut microbiota [17]. Consequently, the therapeutic effects of natural polysaccharides on UC primarily unfold along the axis linking molecular structure, gut microbiota, and the host, achieving dual objectives of controlling inflammation and preserving the intestinal barrier. Specifically, microbial metabolites such as SCFAs can modulate the T helper 17(Th17)/regulatory T-cell (Treg) balance and suppress excessive inflammation by activating pathways involving G protein-coupled receptors (e.g., G protein-coupled receptor 41/43, GPR41/43) [18]. Concurrently, certain metabolites can directly or indirectly inhibit key inflammatory signaling pathways, including NF-κB, mitogen-activated protein kinase (MAPK), and Janus kinase 2 (JAK2)/signal transducer and activator of transcription 3 (STAT3), while enhancing intestinal barrier function and upregulating the expression of tight junction proteins (e.g., zonula occludens-1, ZO-1) and mucin 2 (MUC2) [19]. In summary, through multi-target, multi-pathway synergistic actions, natural polysaccharides demonstrate significant potential in improving gut microecology, attenuating inflammation and mucosal barrier disruption, and alleviating oxidative stress.

Our previous research conceptualizes natural polysaccharides as structure-encoded ecological modulators whose chemical structures guide selective microbial cooperation and metabolic processes, rather than functioning as generic fermentable fibers such as fructooligosaccharides (FOSs) [20]. Existing studies predominantly focus on the pharmacodynamic effects of natural polysaccharides on UC and observations of gut microecological changes. An exploration of the critical mechanistic question of how they exert their intervention effects by driving specific microbial populations—particularly the structure–activity relationship between polysaccharide chemical structures and their microbiota-regulating functions—remains lacking. This review places emphasis on elucidating how natural polysaccharides with distinct structural features specifically reshape gut microbial composition and function, thereby alleviating UC pathology through multiple signaling pathways. It aims to investigate the interaction network and molecular mechanisms by which polysaccharide structures influence the host via the microbiota, with the goal of providing a theoretical basis for developing precise microecological intervention strategies.

## 2. The Framework of Natural Polysaccharides Driving Gut Microbiota to Improve Ulcerative Colitis

Through a broad search of multiple authoritative databases, including PubMed, Web of Science, and Google Scholar, for the literature from the past decade, using core keywords such as “natural polysaccharides,” “gut microbiota,” and “ulcerative colitis,” a preliminary collection of 93 articles was obtained. Following screening, 24 original studies that explicitly reported the detailed structure of polysaccharides (monosaccharide composition, glycosidic linkage types, molecular weight, glycan structure, etc.) and investigated their microbiota-modulating effects were ultimately included for review. Systematic retrieval and dual-person screening were employed, followed by narrative synthesis, descriptive counting, and evidence mapping. This review primarily elaborates on the natural polysaccharides from five aspects: source, monosaccharide composition/proportion and molecular weight, glycan structure, model and therapeutic efficacy, and associated microbial changes, as detailed in Table 1.

The therapeutic action of natural polysaccharides on UC appears to be a precise biological process following the logic of “Structure–Decoding–Conversion–Effect.” This process can be deconstructed into four sequential and interdependent hierarchical levels.

The process begins with the “Structure” of the polysaccharide, which defines the structure-encoded substrate specificity. The chemical structure of a polysaccharide, including its monosaccharide composition and ratios, molecular weight, and the fine conformation of the glycan chain, collectively constitutes a unique carbohydrate signature. This structural signature determines its solubility, viscosity, spatial configuration within the intestinal environment, and its specificity as a substrate for recognition by microbial enzymatic systems. For instance, highly methylated galacturonic acid polymers are preferential substrates for specific polysaccharide utilization loci (PULs) in Bacteroidota [21], whereas β-(1→3)-glucans are readily targeted by glycoside hydrolases from certain Firmicutes bacteria [22].

This structural signature is “Decoding” by the gut microbiome, leading to the Directed Remodeling of the Microbial Community. The specific structure from the first level is recognized by bacteria within the gut microbiome that possess the corresponding repertoire of CAZymes, initiating a cascade of characteristic interactions. The first step is competitive enrichment: bacterial taxa capable of efficiently depolymerizing and utilizing the specific polysaccharide gain a proliferative advantage [23]. As indicated in Table 1, this leads to predictable shifts at the phylum level (e.g., pectin-type polysaccharides enriching Bacteroidota, β-glucans enriching Firmicutes) and directed enrichment of specific functional genera (e.g., butyrate-producing *Lachnospiraceae*_NK4A136_group, lactate-producing *Lactobacillus*, or mucin-regulating *Akkermansia*). This is followed by competitive inhibition: the proliferation of beneficial bacteria alters the intestinal microenvironment (e.g., lowering pH, consuming nutrients, and producing antimicrobial substances) and indirectly suppresses the growth of pro-inflammatory and potentially pathogenic bacteria (e.g., *Escherichia*–*Shigella*, *Helicobacter*) often associated with UC, through mechanisms like colonization resistance [24]. This remodeling is not merely a quantitative change but drives the microbial community towards greater functional redundancy and ecological stability [25].

The stage of “Conversion” constitutes the third level, manifesting as the Hub-like Output of Functional Metabolites. The directionally enriched functional microbiota ferment the polysaccharides into a spectrum of bioactive metabolites, with SCFAs serving as the central hub molecules. Other metabolites, such as secondary bile acids and indole derivatives, may also participate in this process [26], as may the small-molecule oligosaccharides or fragments generated during degradation to some extent [27]. These microbial metabolites constitute the crucial link in the “microbiota–host” crosstalk. The yield and proportion of SCFAs directly reflect the degree of restoration in microbial community function and effectively translate the structural information of the polysaccharide into signals interpretable by host cells [28].

The fourth level is the systemic ameliorative “Effect”, characterized by Multi-Target Amelioration of Host Pathology. The metabolites produced in the third level, particularly SCFAs, act directly on intestinal epithelial cells and gut immune cells as both energy substrates and signaling molecules [29]. They synergistically alleviate the core pathologies of UC through multiple pathways [30]. For example, butyrate, as the preferred energy source for colonic epithelium, promotes the expression of tight junction proteins and mucins by activating pathways such as AMPK, thereby repairing the physical and chemical barrier [31]. SCFAs also modulate the differentiation of key immune cells and inhibit inflammasome signaling pathways like NF-κB by activating G protein-coupled receptors and inhibiting histone deacetylases, consequently reducing pro-inflammatory cytokines and elevating anti-inflammatory cytokine levels [32]. Furthermore, by activating the Nrf2 antioxidant pathway, they enhance the activity of host endogenous antioxidant enzymes such as superoxide dismutase (SOD) and glutathione peroxidase (GSH-Px), mitigating reactive oxygen species (ROS)-mediated tissue damage [33].

**Table 1:** Natural polysaccharides in UC treatment: properties, effects, and microbiota alterations.

Source	Monosaccharide Composition (%) and Mw	Glycan Structure	Model and Efficacy	Microbiota Changes
*Dioscorea opposita* Maxim [34]	Arabinose (0.139), Galactose (0.701), Glucose (83.397), Mannose (15.363), Galacturonic acid (0.401); 832.451 kDa	Backbone: →4)-α-D-Glcp-(1→; →4)-β-D-Man(p)-(1→ Side chain: O-6 of →4,6)-α-D-Glc-(1→: α-D-Glc-(1→; α-D-Glc-(1→6)-α-D-Glc-(1→	Model: DSS-induced UC mice Efficacy: ↑ body weight, ↑ colon length, ↓ disease activity index (DAI); ↓ inflammatory cell infiltration/edema/goblet cell and crypt loss; ↓ serum IL-6 (interleukin-6), TNF-α (tumor necrosis factor-α), IL-17 (interleukin-17); ↑ T-SOD (total superoxide dismutase), CAT (catalase).	Phylum level: Bacteroidota↑; Firmicutes↓, Patescibacteria↓. Family/Genus level: *Bacteroides*↑, *Prevotella*↑, *Phascolarctobacterium*↑; *Lachnospiraceae*↓.
*Thesium chinense* Turcz [35]	Fructose (50), Glucose (37), Galactose (12), Mannose (<1); 5.377 kDa	Backbone: →1)-β-D-Fruf-(2→; →1,6)-β-D-Fruf-(2→; →4)-α-D-Glcp-(1→; →3,6)-β-D-Galp-(1→ Side chain: O-6 of →1,6)-β-D-Fruf-(2→: β-D-Fruf-(2→6)-β-D-Fruf-(2→;O-6 of →3,6)-β-D-Galp-(1→: β-D-Galp-(1→	Model: DSS-induced UC mice Efficacy: ↑ body weight/colon length; improved barrier; ↓ IL-6, TNF-α, IL-17, MDA (malondialdehyde), MPO (myeloperoxidase); ↑ SOD, CAT, IL-10 (interleukin-10).	Phylum level: Bacteroidetes↑; Firmicutes↓. Genus level: *Ruminococcus*↑, *Ligilactobacillus*↑, *Alloprevotella*↑, *Clostridia*_UCG-014↑, *Bifidobacterium*↑; *Helicobacter*↓, *Adlercreutzia*↓, *Desulfovibrio*↓, *Parasutterella*↓.
*Hericium erinaceus* [36]	Glucose (8.53), Mannose (90.15), Galactose (1.33); 3.1 kDa	Backbone: α-D-Glc(1→3); β-D-Glc(1→3) Side chain: O-4 of →3,4)-Glcp-(1→: Manp-(1→; Glcp-(1→;O-4 (or O-3) of →3,4)-Galp-(1→: Glcp/Manp-(1→	Model: Acetic acid-induced UC rats Efficacy: ↑ body weight; improved colon morphology/inflammation; ulcer healing; ↓ IL-1 (interleukin-1), IL-6, MDA; ↑ SOD; ↑ IgM (immunoglobulin M), C3 (complement component 3).	Phylum level: Proteobacteria↓. Family/Genus level: *Ruminococcaceae*↑, *Lachnospiraceae*↑, *Akkermansia*↑, *Allobaculum*↑, *Desulfovibrionales*↑.
Barley bran [37]	Xylose (48.87), Arabinose (37.13), Glucose (8.53), Galactose (5.47); 227.6 kDa	Backbone: β-1,4-linked xylose Side chain: O-2 of →4)-β-D-Xylp-(1→: →4)-β-D-Xylp-(1→ via α-L-Araf-(1→; O-3 of →4)-β-D-Xylp-(1→: →4)-β-D-Xylp-(1→ via α-L-Araf-(1→; O-2,3 of →4)-β-D-Xylp-(1→: →4)-β-D-Xylp-(1→ via α-L-Araf-(1→ at O-2 and O-3	Model: DSS-induced UC mice Efficacy: ↑ body weight/colon length, ↓ DAI; ↓ inflammation/oxidative stress; ↑ tight-junction proteins and mucins; ↑ goblet cells/mucus.	Phylum level: Actinobacteria↑; Proteobacteria↓. Family/Genus level: *Ligilactobacillus*↑, *Lachnospiraceae*_NK4A136_group↑; *Escherichia*–*Shigella*↓, *Helicobacter*↓.
*Asimina triloba* [38]	Mannose (2.23), Rhamnose (6.43), Glucose (50.12), Galactose (8.36), Arabinose (31.91); 164.504 kDa	Backbone: →4)-α-D-Glcp-(1→ Side chain: O-3 of →3)-α-L-Rhap-(1→: →4)-α-D-Glcp-(1→; O-4 of →4)-α-D-Glcp-(1→: →4,6)-β-D-Galp-(1→; O-6 of →4,6)-β-D-Galp-(1→: →3,6)-β-D-Manp-(1→; O-4 of →4,6)-β-D-Galp-(1→: α-D-Glcp-(1→; O-6 of →3,6)-β-D-Manp-(1→: →2,5)-α-L-Araf-(1→; O-2 of →2,5)-α-L-Araf-(1→: α-L-Araf-(1→;O-5 of →2,5)-α-L-Araf-(1→: →5)-α-L-Araf-(1→	Model: DSS-induced UC mice Efficacy: ↑ body weight/colon length, ↓ DAI; ↓ tissue damage; ↑ goblet cells; ↑ claudin-1, ZO-1, occludin; ↓ IL-6, TNF-α; ↑ IL-10.	Phylum level: Bacteroidetes↑; Firmicutes↓, Proteobacteria↓. Genus level: *Lactobacillus*↑, *Muribaculum*↑, *Bifidobacterium*↑, *Turicobacter*↑; *Enterobacteriaceae*↓.
*Lycium barbarum* L. [39]	Arabinose (43.84), Galactose (20.42), Glucose (3.90), Galacturonic acid (24.67), Rhamnose (6.20), Mannose (0.97); 102.2 kDa	Backbone: →2)-α-L-Rhap-(1→4)-α-D-GalAp-(1→6)-β-D-Galp-(1→ Side chain: O-3 of →3,6)-β-D-Galp-(1→: [α-L-Araf-(1→), →5)-α-L-Araf-(1→), →3,5)-α-L-Araf-(1→), β-D-Galp-(1→), →3)-β-D-Galp-(1→)]; O-2 of →2,4)-α-L-Rhap-(1→: [α-L-Araf-(1→), →5)-α-L-Araf-(1→), →3,5)-α-L-Araf-(1→), β-D-Galp-(1→), →3)-β-D-Galp-(1→)]; O-4 of →2,4)-α-L-Rhap-(1→: [α-L-Araf-(1→), →5)-α-L-Araf-(1→), →3,5)-α-L-Araf-(1→), β-D-Galp-(1→), →3)-β-D-Galp-(1→)]	Model: DSS-induced UC mice Efficacy: ↑ body weight/colon length, ↓ DAI; ↓ ulcer area/crypt damage.	Phylum level: Firmicutes↑; Bacteroidetes↓, Proteobacteria↓. Genus level: *Muribaculaceae*↑, *Lactobacillus*↑, *Lachnospiraceae*_NK4A136_group↑; *Bacteroides*↓, *Escherichia*–*Shigella*↓, *Dubosiella*↓, *Faecalibaculum*↓, *Clostridium*_sensu_stricto_1↓.
*Porphyra haitanensis* [40]	Galactose; 9.7 kDa	Backbone: →3)-β-D-Galp-(1→4)-α-L-Galp-6S-(1→; →3)-β-D-Galp-(1→4)-3,6-anhydro-α-L-Galp-(1→	Model: DSS-induced UC mice Efficacy: ↑ body weight/colon length, ↓ DAI; restored mucosa; ↓ inflammatory infiltration; ↑ mucus thickness; ↑ tight-junction proteins; ↓ CD4^+^/CD8^+^ T-cell infiltration.	Phylum level: Bacteroidetes↑; Firmicutes↓. Genus level: *Bacteroides*↑, *Muribaculum*↑, *Lactobacillus*↑.
*Astragalus membranaceus* [41]	Mannose (-), Rhamnose (-), Galacturonic acid (-), Glucose (-), Galactose (-), Arabinose (-); 104.9 kDa	Backbone: →6)-β-Galp-(1→4)-β-Galp-(1→	Model: DSS-induced UC mice Efficacy: ↑ body weight/colon length, ↓ DAI; ↓ inflammatory infiltration/crypt deformation/mucus damage; ↑ tight-junction proteins.	Phylum level: Proteobacteria↓. Family/Genus level: *Muribaculaceae*↑, *Lachnospiraceae*↑, *Rikenellaceae*↑, *Ruminococcaceae*↑, *Prevotellaceae*↑; *Bacteroides*↓.
Flower Mushroom [42]	Glucose; 720 kDa	Backbone: (1→3)-β-D-Glcp	Model: DSS-induced UC mice Efficacy: ↓ DAI/colon bleeding; ↑ occludin, ZO-1, MUC2 (mucin-2); ↑ goblet cells; ↓ TNF-α, IL-1β (interleukin-1β), IL-6, IL-17A (interleukin-17A); restored Th17/Treg balance.	Phylum level: Firmicutes↑; Bacteroidetes↓, Proteobacteria↓. Family/Genus level: *Lachnospiraceae*_NK4A136_group↑, *Clostridia*↑, *Odoribacter*↑; *Bacteroides*↓, *Helicobacter*↓, *Parasutterella*↓, *Romboutsia*↓, *Oscillibacter*↓.
*Grifola frondosa* [43]	Glucose (86.9), Galactose (6.3), Mannose (3.8), Fucose (2.5); 256.5 kDa	Backbone: →4)-Glcp-(1→, →4)-Galp-(1→; →3,6)-Mann-(1→, →4,6)-Galp-(1→ Side chain: O-6 of →1,4,6)-β-D-Galp-(1→: β-D-Glcp-(1→; →4)-β-D-Glcp-(1→; α-L-Fucp-(1→3)- O-3 and/or O-6 of →1,3,6)-α-D-Manp-(1→: β-D-Glcp-(1→; →6)-β-D-Glcp-(1→; α-L-Fucp-(1→3)	Model: Oxazolone-induced UC mice Efficacy: ↓ DAI/colon shortening; ↑ goblet cells/mucus; ↓ fibrosis; ↑ claudin-1, ZO-1; ↑ PCNA (proliferating cell nuclear antigen); ↑ SOD, CAT, GSH-Px (glutathione peroxidase); ↓ MDA.	Phylum level: Firmicutes↑, Desulfobacterota↑, Deferribacterota↑; Bacteroidota↓, Proteobacteria↓, Patescibacteria↓. Family/Genus level: *Lactobacillus*↑, *Lachnospiraceae*↑, *Roseburia*↑; *Escherichia*–*Shigella*↓, *Muribaculaceae*↓, *Bacteroides*↓.
*Tremella aurantialba* [44]	Mannose (59.2), Xylose (23.2), Glucuronic acid (13.9), Glucose (1.6), Fucose (1.7), Rhamnose (0.4); 127 kDa	Backbone: →3)-Manp-(1→ Side chain: O-2 of →1,3)-α-D-Manp-(1→: β-D-Xylp-(1→; →1,3)-β-D-Xylp-(1→; →1,4)-β-D-GlcAp-(1→; α-D-Manp-(1→; β-D-Glcp-(1→	Model: DSS-induced UC mice Efficacy: ↑ survival; ↓ weight loss/colon shortening; ↓ IL-6, TNF-α, MCP-1 (monocyte chemoattractant protein-1); ↓ histopathology damage; ↑ claudin-1, ZO-1.	Phylum level: Firmicutes↑, Verrucomicrobia↑; Bacteroidetes↓. Genus level: *Akkermansia*↑, *Adlercreutzia*↑, *Lactobacillus*↑, *Bifidobacterium*↑; *Bacteroides*↓, *Ruminococcus*↓.
*Laminaria japonica* [45]	Guluronic acid (91.86), Mannuronic acid (8.14); 8.59 kDa	Backbone: →1,4)-α-L-GulAp-(1→; →1,4)-β-D-ManAp-(1→	Model: DSS-induced UC mice Efficacy: ↓ weight loss/colon shortening/bleeding; ↓ mucosal damage; ↓ inflammatory infiltration; ↑ mucus O-glycan synthesis.	Phylum level: Firmicutes↑; Bacteroidetes↓. Family/Genus level: *Lactobacillus*↑, *Muribaculaceae*↑, *Prevotellaceae*↑; *Bacteroides*↓, *Alistipes*↓.
*Diospyros lotus* L. [46]	Galacturonic acid (75.49), Rhamnose (3.32), Galactose (9.86), Arabinose (7), Xylose (4.32), Mannose (<1), Glucose(<1); 153.95 kDa	Backbone: →4)-α-GalpA-(1→; →3,4)-GalpA-(1→; →2,4)-GalpA-(1→ Side chain: O-4 of →2)-α-L-Rhap-(1→: α-L-Araf-(1→; →5)-α-L-Araf-(1→; β-D-Galp-(1→; O-3/O-4 of →4)-α-D-GalpA-(1→: β-D-Xylp-(1→	Model: DSS-induced UC mice Efficacy:↓ weight loss/DAI; improved colon shortening/splenomegaly; ↓ histopathology damage; ↑ claudin-1, occludin; ↓ IL-1β, IL-6, TNF-α, MPO, MDA; ↑ IL-10.	Phylum level: Firmicutes↑, Bacteroidetes↑; Proteobacteria↓. Family/Genus level: *Lachnospiraceae*↑, *Lactobacillaceae*↑; *Enterobacteriaceae*↓.
*Citrus medica* [47]	Rhamnose (1.57), Arabinose (4.46), Galactose (2.50), Galacturonic acid (91.47); 38.28 kDa	Backbone: →4)-α-D-GalAp-6-O-CH_3_-(1→3,4)-α-D-GalAp-6-O-CH_3_ Side chain: O-3 of →4)-α-D-GalAp-6-O-Me-(1→: α-D-GalAp-(1→; O-4 of →2)-α-L-Rhap-(1→: α-L-Araf-(1→; β-D-Galp-(1→	Model: DSS-induced UC mice Efficacy: ↓ weight loss/DAI; ↑ colon length; ↓ histopathology damage/inflammatory markers; ↑ barrier proteins.	Phylum level: Bacteroidetes↑, Verrucomicrobiota↑; Firmicutes↓. Family/Genus level: *Muribaculaceae*↑, *Clostridia*↑, *Lachnospiraceae*↑, *Alistipes*↑, *Butyricimonas*↑; *Dubosiella*↓, *Klebsiella*↓, *Peptostreptococcaceae*↓, *Escherichia*–*Shigella*↓, *Mucispirillum*↓.
*Aloe* [48]	Mannose (84.2), Glucose (7.2), Galactose (4.6), Rhamnose (2.2), Uronic acid (1.8); 78.8 kDa	Backbone: →4)-β-Manp-(1→ Side chain: O-3 of →4)-β-D-Manp-(1→: β-D-Manp-(1→(→3)-Manp-(1→); →4)-β-D-Glcp-(1→	Model: DSS-induced UC mice Efficacy: ↓ weight loss/colon shortening/DAI; ↓ pro-inflammatory cytokines; ↑ anti-inflammatory cytokines and tight-junction proteins; ↑ serum antioxidant enzymes.	Phylum level: Firmicutes↑; Bacteroidetes↓, Proteobacteria↓. Genus level: *Akkermansia*↑, *Turicobacter*↑; *Parabacteroides*↓, *Anaerostipes*↓, *Blautia*↓, *Escherichia*–*Shigella*↓.
*Cyclocarya paliurus* [49]	Glucose (31.8), Galactose (27.9), Arabinose (17.6), Galacturonic acid (14.2), Mannose (8.4); 900 kDa	Backbone: →4)-α-D-GalpA-(1→; →2)-α-L-Rhap-(1→4)-α-D-GalpA-(1→	Model: DSS-induced UC mice Efficacy: ↓ weight loss, ↑ colon length; ↓ MPO and inflammatory factors; ↑ IL-10; ↑ tight-junction proteins; ↓ serum ET (endotoxin) and LBP (lipopolysaccharide-binding protein); ↑ hepatic SOD, GSH-Px, CAT; ↓ MDA.	Phylum level: Bacteroidetes↑; Firmicutes↓, Verrucomicrobiota↓, Proteobacteria↓. Genus level: *Alistipes*↑, *Sphingomonas*↑, *Lactobacillus*↑, *Coprococcus*↑; *Akkermansia*↓, *Clostridium*↓, *Helicobacter*↓, *Prevotella*↓, *Sutterella*↓.
*Dioscorea opposita* Maxim [49]	Galactose (28.57), Glucose (11.28), Galacturonic acid (37.59); 20.89 kDa	Backbone: →3)-β-D-Glcp-(1→; →6)-β-D-Galp-(1→ Side chain: 1,3-linked-Glc, 1,6-linked-Gal, 1-linked-Gal	Model: DSS-induced UC mice Efficacy: ↓ weight loss; ↑ colon length; ↓ MPO and inflammatory factors; ↑ tight-junction proteins; ↓ serum ET, LBP; ↑ hepatic SOD, GSH-Px, CAT; ↓ MDA.	Phylum level: Bacteroidetes↑; Firmicutes↓, Verrucomicrobiota↓, Proteobacteria↓. Genus level: *Bacteroides*↑, *Mucispirillum*↑, *Lactobacillus*↑, *Coprococcus*↑; *Akkermansia*↓, *Clostridium*↓, *Helicobacter*↓, *Prevotella*↓, *Sutterella*↓.
*Zingiber officinale* Roscoe [50]	Glucose (85.09), Galactose (5.00), Arabinose (9.91); 83.72 kDa	Backbone: →4,6)-β-Glcp-1→; →3,6)-α-Galp-1→ Side chain: O-6 of →4,6)-β-D-Glcp-(1→: α-Araf-(1→4)-β-D-Glcp-(1→; O-6 of →3,6)-α-D-Galp-(1→: α-Araf-(1→4)-β-D-Glcp-(1→	Model: DSS-induced UC mice Efficacy:↓ weight loss/diarrhea/hematochezia/DAI; ↓ colon shortening/thickening and mucosal damage; ↓ inflammatory infiltration; ↓ spleen index, ↑ thymus index; ↓ inflammatory factors; ↑ tight-junction proteins.	Phylum level: Firmicutes↑, Actinobacteria↑, Verrucomicrobia↑; Bacteroidetes↓, Proteobacteria↓. Family/Genus level: *Enterococcaceae*↓, *Erysipelotrichaceae*↓.
*Gastrodia elata* Blume [51]	Glucose (>99), Mannose(<1); 811.0 kDa	Backbone: →4)-α-D-Glcp(1→ Side chain: O-6 of →4)-α-D-Glcp-(1→: α-D-Glcp-(1→	Model: DSS-induced UC mice Efficacy: ↓ weight loss, ↑ colon length, ↓ DAI; ↓ histopathology damage; ↑ goblet cells; ↓ inflammatory factors; ↓ NLRP3 (NOD-like receptor protein 3) and ASC (apoptosis-associated speck-like protein containing a CARD); ↑ MUC2; ↑ ZO-1, occludin.	Phylum level: Firmicutes↑. Genus level: *Bacteroides*↓, *Butyricimonas*↓, *Alistipes*↓.
*Ishige okamurae* [52]	Fucose (75.0), Mannose (9.1), Galactose (8.5), Glucose (4.5), Xylose (3.1); [-]	Backbone: →3)-α-L-Fucp-(1→; →4)-α-L-Fucp-(1→ Side chain: O-2 of →2,4)-α-L-Fucp-(1→: t-β-D-Xylp-(1→; O-3 of →3,4)-α-L-Fucp-(1→: t-β-D-Glcp-(1→; O-6 of →6)-β-D-Galp-(1→: β-D-Galp-(1→; O-2 of →2)-β-D-Manp-(1→: β-D-Manp-(1→	Model: DSS-induced UC mice Efficacy: ↓ DAI/diarrhea/hematochezia; ↑ colon length; ↓ mucosal ulcers and inflammatory infiltration; ↑ goblet cells/mucus; ↓ inflammatory factors; ↑ ZO-1, occludin, claudin-1.	Phylum level: Bacteroidota↑, Campilobacterota↑; Firmicutes↓, Actinobacteria↓. Genus level: *Rikenella*↑; *Dubosiella*↓, *Romboutsia*↓, *Escherichia*–*Shigella*↓, *Desulfovibrio*↓.
*Areca catechu* L. [53]	Galactose (40.1), Mannose (32.4), Arabinose (25.2), Glucose (2.3); 65 kDa	Backbone: →3,6)-β-Gal-(1→; →2,4,6)-β-Man-(1→ Side chain: O-3 of →3,6)-β-D-Galp-(1→: α-L-Araf-(1→; O-6 of →4,6)-β-D-Glcp-(1→: β-D-Glcp-(1→; O-2 of →2)-β-D-Glcp-(1→: β-D-Glcp-(1→; O-6 of →2,6)-β-D-Manp-(1→: β-D-Glcp-(1→; O-2 of →2,6)-β-D-Manp-(1→: β-D-Glcp-(1→; O-6 of →3,6)-β-D-Galp-(1→: β-D-Galp-(1→	Model: DSS-induced UC mice Efficacy:↓ weight loss/colon shortening/DAI; ↓ pro-inflammatory factors; ↑ tight-junction proteins; improved histology.	Phylum level: Bacteroidota↑; Firmicutes↓. Genus level: *Ligilactobacillus*↑, *Dubosiella*↑, *Lactobacillus*↑, *Ruminococcus*↑; *Bacteroides*↓, *Parasutterella*↓, *Alistipes*↓.
*Sagittaria sagittifolia* L. [54]	Glucose (81.37), Arabinose (5.85), Mannose (7.52), Galactose (5.26); 65.79 kDa	Backbone: →4)-Glcp-(1→; →6)-Glcp-(1→ Side chain: O-3 of →3,6)-β-D-Galp-(1→: α-L-Araf-(1→; O-3 of →3,6)-β-D-Galp-(1→: →5)-α-L-Araf-(1→; O-2 of →2,4,6)-β-D-Manp-(1→: β-D-Galp-(1→	Model: DSS-induced UC mice Efficacy:↓ DAI/weight loss; ↑ colon length; ↓ inflammatory factors; ↑ SOD, ↓ MDA; ↑ claudin-1, occludin, ZO-1.	Phylum level: Bacteroidetes↑; Firmicutes↓, Verrucomicrobiota↓, Proteobacteria↓, Actinobacteria↓. Genus level: *Lactobacillus*↑, *Candidatus*_*Saccharimonas*↑, *Nitrosospira*↑, *Dialister*↑; *Alistipes*↓, *Akkermansia*↓, *Ligilactobacillus*↓, *Limosilactobacillus*↓, *Pseudomonas*↓.
*Bletilla striata* [55]	Mannose (70.68), Glucose (29.32); 78.15 kDa	Backbone: →4)-β-D-Manp-(1→; →4)-β-D-Glcp-(1→ Side chain: O-3 of →1,4)-β-D-Glcp-(1→: β-D-Glcp-(1→; O-3 of →1,4)-β-D-Galp-(1→: β-D-Galp-(1→;O-2 of →1,4)-β-D-Galp-(1→: β-D-Galp-(1→;O-6 of →1,4)-β-D-Galp-(1→: β-D-Galp-(1→;O-3 of →1,4)-β-D-Galp-(1→: α-L-Araf-(1→;O-6 of →1,4)-β-D-Galp-(1→: α-L-Araf-(1→5)-α-L-Araf-(1→	Model: DSS-induced UC mice Efficacy: ↓ weight loss/DAI/colon shortening; ↓ pro-inflammatory factors; ↑ anti-inflammatory factors; ↑ SOD, ↓ MDA; ↑ occludin, ZO-1, claudin-1, MUC2; ↓ serum DAO (diamine oxidase).	Phylum level: Firmicutes↑, Bacteroidota↑, Actinobacteria↑; Proteobacteria↓. Family/Genus level: *Lactobacillus*↑; *Escherichia*–*Shigella*↓.
*Phyllostachys edulis* [56]	Arabinose (10.09), Glucose (63.14), Galactose (26.77); 80.8 kDa	Backbone: β-(1→4)-Glcp, →3)-Glcp-(1→, →4)-Galp-(1→ Side chain: O-3 of →1,4)-β-D-Manp-(1→: β-D-Manp-(1→;O-2 of →1,4)-β-D-Glcp-(1→: β-D-Manp-(1→;O-2 of →1,4)-β-D-Manp-(1→	Model: DSS-induced UC mice Efficacy: ↑ body weight, ↓ DAI, ↓ colon shortening; ↓ edema/inflammation/histologic injury; ↑ ZO-1, claudin-1, occludin; ↓ inflammatory markers.	Phylum level: Firmicutes↑; Bacteroidetes↓, Proteobacteria↓, Deferribacteres↓. Genus level: *Prevotella*↑, *Alistipes*↑, *Anaerostipes*↑, *Odoribacter*↑, *Bifidobacterium*↑, *Butyricimonas*↑, *Lactobacillus*↑; *Parabacteroides*↓, *Mucispirillum*↓, *Helicobacter*↓, *Bacteroides*↓, *Streptococcus*↓.

Glcp: D-glucopyranose; Manp: D-mannopyranose; Galp: D-galactopyranose; GalAp: D-galacturonic acid; GlcAp: D-glucuronic acid; Fruf: D-fructofuranose; Araf: L-arabinofuranose; Rhap: L-rhamnopyranose; Fucp: L-fucopyranose; Xylp: D-xylopyranose; and ManA: D-mannuronic acid. Linkage numbers indicate glycosidic positions; “→” indicates linkage direction. Arrows denote direction of change relative to the disease control (↑ increased; ↓ decreased).

## 3. Structural Specificity of Natural Polysaccharides in UC Studies

**Figure 1:**
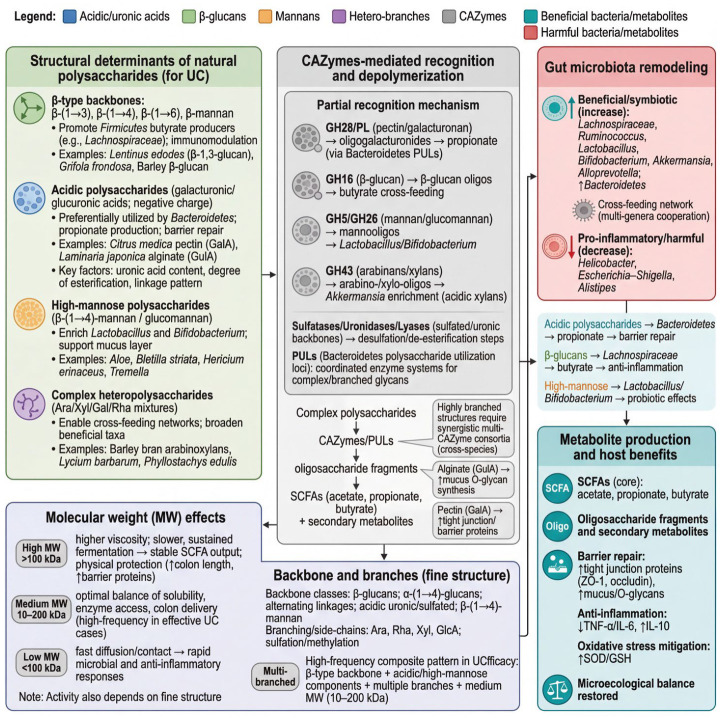
Cascade process of structural specificity of natural polysaccharides driving gut microbiota remodeling and metabolite generation. Abbreviations: UC: ulcerative colitis; CAZymes: carbohydrate-active enzymes; PULs: polysaccharide utilization loci (Bacteroidetes polysaccharide utilization systems); SCFAs: short-chain fatty acids; GH: glycoside hydrolase (e.g., GH16, GH28, GH5, GH26, and GH43 family numbers); PL: polysaccharide lyase (e.g., pectate/pectin lyases); Ara: arabinose; Xyl: xylose; Gal: galactose; Rha: rhamnose; GalA: galacturonic acid; GlcA: glucuronic acid; GulA: guluronic acid; ZO-1: zonula occludens-1; SOD: superoxide dismutase; GSH: glutathione; TNF-α: tumor necrosis factor alpha; IL-6: interleukin-6; IL-10: interleukin-10; MW: molecular weight; O-glycans: O-linked glycans; and β-glucans: beta-glucans. Green arrows indicate upregulation, and red arrows indicate downregulation.

The interventive efficacy of natural polysaccharides in UC is not a simple manifestation of their natural attributes but is highly dependent on their complex chemical structures [57]. The bioactivity of polysaccharides is closely related to their molecular architecture, including monosaccharide composition and ratio, molecular weight, glycosidic bond types, and linkage patterns, among other factors [58]. These structural parameters collectively determine the specificity of polysaccharides as substrates for microbial fermentation, thereby ultimately influencing their ability to remodel the gut microbiota and modulate host inflammation [59]; please see Figure 1.

### 3.1. Microbial Enrichment Specificity Appears Linked to Monosaccharide Composition

The types and ratios of monosaccharides and the content of acidic groups are the core chemical characteristics that determine the physicochemical properties of polysaccharides and microbial utilization preferences, which are typically categorized into acidic polysaccharides and neutral polysaccharides [60]. Different bacterial taxa within the gut microbiota possess specific repertoires of CAZymes, enabling them to specifically recognize and degrade structures composed of particular monosaccharide units [61].

#### 3.1.1. Acidic Polysaccharides

Polysaccharides rich in acidic monosaccharides such as galacturonic acid and glucuronic acid (e.g., from *Lycium barbarum*, *Citrus medica*, *Diospyros lotus*, *Cyclocarya paliurus*, and *Laminaria japonica*) typically carry a negative charge. Their bioactivity is closely related to the content of uronic acids, the degree of esterification, and linkage patterns. This type of acidic polysaccharide is the preferred substrate for Bacteroidetes [62]. Our evidence mapping shows that the vast majority of acidic polysaccharide interventions significantly increased the abundance of Bacteroidetes. After fermentation, they mainly produce SCFAs like propionate, which are associated with anti-inflammatory effects and repair of intestinal barrier function. For example, *Laminaria japonica* (a polymer of guluronic acid) can increase O-glycan synthesis in the mucus layer, while *Citrus medica* pectin (a polymer of galacturonic acid) can enhance the expression of barrier proteins.

#### 3.1.2. Neutral Polysaccharides

Firstly, glucose-dominant polysaccharides refer to neutral glucans where glucose constitutes the backbone or main component (e.g., from Flower mushroom, *Grifola frondosa*, *Gastrodia elata* Blume, and *Zingiber officinale* Roscoe). Their activity highly depends on the type of glycosidic bonds (β-1,3/1,4/1/6, etc.). Classic β-glucans have well-defined immunomodulatory functions. These polysaccharides often promote the growth of butyrate-producing genera within the Firmicutes phylum, thereby enhancing anti-inflammatory and barrier-repair effects [63].

Secondly, mannose-dominant polysaccharides refer to polysaccharides with high mannose content (e.g., from *Hericium erinaceus*, *Aloe*, *Bletilla striata*, and *Tremella aurantialba*). These often specifically promote the proliferation of *Lactobacillus* and *Bifidobacterium*. The mechanism of action may be related to serving as a specific carbon source for probiotics, or it may interfere with pathogen colonization through molecular mimicry [64].

Finally, complex monosaccharide-type polysaccharides refer to complex heteropolysaccharides composed of multiple monosaccharides such as arabinose, xylose, galactose, and rhamnose in specific proportions (e.g., from Barley bran, *Lycium barbarum*, and *Phyllostachys edulis*). Their structural complexity mimics the diversity of dietary fibers. These polysaccharides can support a broader microbial cross-feeding network, simultaneously upregulating multiple beneficial genera, achieving more comprehensive microbiota homeostasis regulation and metabolite output [65].

### 3.2. Molecular Weight Affects the Physical Properties and Behavioral Patterns of Polysaccharides in the Intestine

Molecular weight determines the physical form of polysaccharides in the intestine, their contact efficiency with microorganisms, and fermentation kinetics by influencing solubility, viscosity, diffusion rate, and enzymatic susceptibility [66].

#### 3.2.1. High-Molecular-Weight Polysaccharides

A molecular weight greater than 100 kDa is generally considered high molecular weight, such as *Dioscorea opposita* Maxim polysaccharide (832.5 kDa), *Gastrodia elata* Blume polysaccharide (811 kDa), Flower mushroom polysaccharide (720 kDa), etc. High molecular weight typically leads to higher solution viscosity, enabling the formation of a physical barrier within the intestine, potentially delaying gastric emptying and small intestinal absorption, allowing more to reach the distal gut. The fermentation process of high-molecular-weight polysaccharides is usually slower and more prolonged, favoring the production of a sustained and stable flow of SCFAs and providing long-lasting physical protection [67]. In our evidence mapping, most high-molecular-weight polysaccharides showed good effects on body weight recovery, colon length recovery, and upregulation of barrier proteins.

#### 3.2.2. Medium- and Low-Molecular-Weight Polysaccharides

A molecular weight less than 100 kDa is generally considered medium or low molecular weight, such as *Thesium chinense* polysaccharide (5.4 kDa), *Hericium erinaceus* polysaccharide (3.1 kDa), *Porphyra haitanensis* polysaccharide (9.7 kDa), etc. Lower molecular weight means better solubility and higher diffusion rates, allowing them to be contacted and utilized by the gut microbiota more quickly and extensively, thereby initiating faster microbial modulation and anti-inflammatory responses [68]. For example, low-molecular-weight *Thesium chinense* polysaccharide can rapidly downregulate pro-inflammatory factors and upregulate antioxidant enzymes.

More often, the effect of molecular weight is closely intertwined with structure [69]. For instance, low-molecular-weight *Laminaria japonica* (8.6 kDa) possesses specific activity in promoting mucus secretion due to its unique guluronic acid sequence, while the potent effects of some high-molecular-weight polysaccharides may also stem from their unique immunomodulatory conformations. Therefore, molecular weight is an important parameter for assessing polysaccharide release rate, but the ultimate activity often depends on the chemical structure itself.

### 3.3. Glycan Structure Appears to Be a Primary Determinant of Functional Specificity

The fine structure of glycans, including the position and type of glycosidic linkages (α/β) in the backbone, degree of branching, side-chain monosaccharide sequence, and modifications like sulfation/methylation, is the core of specific recognition by microbial enzyme systems and the triggering of different immunomodulatory pathways [70].

#### 3.3.1. Backbone Linkage Patterns

The type of glycosidic bonds in the backbone is key to determining the rigidity of the polysaccharide skeleton and the ease of enzymatic degradation [71]. According to table evidence mapping, the backbone linkage patterns of polysaccharides for UC treatment can be summarized into the following categories based on their chemical features and functional attributes: The first category is β-type glucans. For example, Flower mushroom polysaccharide has a backbone of (1→3)-β-D-Glcp; *Grifola frondosa*, *Phyllostachys edulis*, and Barley bran polysaccharides contain β-(1→4) linkages. These structures typically exhibit strong bioactivity and resistance to enzymatic degradation. The second category is α-type-linked starch-like polysaccharide backbones. For example, *Asimina triloba*, *Gastrodia elata* Blume, and *Dioscorea opposita* Maxim polysaccharides contain →4)-α-D-Glcp-(1→ backbones, which are easily recognized and decomposed by amylases, and their metabolic pathways are relatively clear. The third category is complex alternating linkage backbones, which are usually composed of two or more types of monosaccharide residues alternating, with high structural complexity requiring specific synergistic enzyme systems for deconstruction, for example, *Thesium chinense* polysaccharide, with alternating fructose and glucose (→1)-β-D-Fruf-(2→ with →4)-α-D-Glcp-(1→), *Hericium erinaceus* polysaccharide, with alternating α- and β-glucose (α-D-Glc(1→3) and β-D-Glc(1→3)), etc. The fourth category is acidic backbones rich in uronic acids or sulfate groups. These polysaccharides require uronidases, sulfatases, or specific lyases for degradation. This includes sulfated structures like →3)-β-D-Galp-(1→4)-α-L-Galp-6S-(1→ in *Porphyra haitanensis* polysaccharide, and highly esterified backbones based on α-D-GalAp found in *Diospyros lotus*, *Citrus medica*, and other polysaccharides. Additionally, some polysaccharides have hybrid or highly branched backbones, such as *Lycium barbarum* polysaccharide with →2)-α-L-Rhap-(1→4)-α-D-GalAp-(1→6)-β-D-Galp-(1→ and a similar structure in *Cyclocarya paliurus* polysaccharide (→4)-α-D-GalpA-(1→, →2)-α-L-Rhap-(1→4)-α-D-GalpA-(1→). Their complex glycosidic bond arrangements further enhance structural stability and functional specificity. Finally, polysaccharides with β-(1→4)-mannose backbones (e.g., →4)-β-Manp-(1→ in *Aloe* and *Bletilla striata* polysaccharides) are also found in various sources, showing unique roles in maintaining the intestinal mucus layer and regulating microbiota.

#### 3.3.2. Branching and Side-Chain Structure

The complexity of branching greatly expands polysaccharide functionality. The type, linkage pattern, and substitution site of side-chain monosaccharides not only provide diverse microbial recognition sites but also finely guide the attachment, enzymatic cleavage, and utilization patterns of specific microbial groups [72]. For example, the xylose and glucuronic acid side chains in the *Tremella aurantialba* polysaccharide may be closely related to its significant upregulation of *Akkermansia* abundance, as mucus-degrading bacteria like *Akkermansia* are typically rich in CAZymes that can utilize acidic xylans. Similarly, the abundant arabinose (T-Araf, 1,5-Araf) and rhamnose (1,3-Rhap) units in the side chains of *Asimina triloba* polysaccharide likely attract specific microbial communities in the gut capable of degrading pectin side chains, consistent with the observed upregulation trend of beneficial bacteria like *Lactobacillus* in experiments. The unique fructose backbone and side chains of *Thesium chinense* polysaccharide and the arabinose side chains of Barley bran polysaccharide provide unique substrates for specific microbial groups, which may explain the enrichment effect of Barley bran polysaccharide on genera with arabinose utilization capacity, like *Ligilactobacillus* and *Lachnospiraceae*_NK4A136_group.

Highly branched polysaccharide structures with diverse linkage sites impose higher demands on microbial degradative capabilities, often requiring synergistic action of multiple CAZymes [73]. For example, *Lycium barbarum* polysaccharide has a backbone composed of rhamnose, galacturonic acid, and galactose, along with arabinose side chains connected at multiple sites (e.g., →3,5)-α-L-Araf-(1→). This complex structure is nearly impossible for a single bacterial strain to completely decompose. Its degradation likely requires a consortium of different species working together, promoting microbial mutualism and helping to build a stable microbial cross-feeding network, thereby amplifying its prebiotic effect. Similarly, *Grifola frondosa* polysaccharide’s backbone contains linkages like →4)-Glcp, →4)-Galp, and →3,6)-Mann, and side chains include fucose (→3)-Fucp-(1→), pointing to a similar synergistic degradation pattern due to its complex structure.

Whether a polysaccharide can simultaneously and broadly modulate multiple beneficial genera is often directly related to the diversity of its side chains [74]. Taking *Thesium chinense* polysaccharide as an example, it not only upregulates *Ruminococcus* (capable of degrading β-linked polysaccharides) but also enriches multiple genera, including *Ligilactobacillus* (prefers fermenting sugars), *Bifidobacterium* (can utilize complex carbohydrates), and *Alloprevotella*. This effect of “one polysaccharide benefiting multiple bacteria” is likely attributed to the simultaneous presence in its structure of fructose and galactose branches composed of various linkage patterns (e.g., →1,6)-β-D-Fruf-(2→, →3,6)-β-D-Galp-(1→), providing diverse “food” options for bacteria with different metabolic preferences.

The linkage patterns of side chains also show fine regulatory effects. For example, *Aloe* and *Bletilla striata* polysaccharides, both with β-(1→4)-mannose backbones, may have different final regulatory impacts on microbiota profiles due to differences in side-chain linkages (e.g., the →3)-Manp-(1→ side chain in *Aloe* polysaccharide). *Aloe* polysaccharide significantly upregulated *Akkermansia* and *Turicibacter*, while *Bletilla striata* polysaccharide mainly upregulated *Lactobacillus* and *Muribaculaceae*. This indicates that even with identical backbones, subtle differences in side chains can ultimately guide the formation of different gut microbiota ecologies by affecting the accessibility and specificity of enzymatic cleavage sites.

### 3.4. Structural Specificity of Polysaccharides in UC Treatment

Based on evidence mapping of structural and efficacy data from 24 polysaccharides in the table, polysaccharides effective in UC treatment show the following high-frequency structural tendencies: backbone with β-type linkages (especially β-(1→3), β-(1→4), or β-mannan), which are present in 20 polysaccharides and clearly associated with promoting butyrate-producing bacteria (e.g., *Lachnospiraceae*) and immunomodulation. Acidic features containing uronic acids (galacturonic/glucuronic acid)—a major component in 12 polysaccharides—are consistently accompanied by increased Bacteroidetes abundance and barrier repair. Mannose content >15%, which is found in 10 polysaccharides, universally causes significant proliferation of *Lactobacillus* or *Bifidobacterium*. Molecular weight between 10 kDa and 200 kDa covers 16 polysaccharides, balancing solubility, enzymatic accessibility, and colon delivery efficiency. The presence of side chains (especially containing heterogeneous monosaccharides like arabinose, xylose, and fucose) is found in 21 polysaccharides; their complexity is closely related to synergistic enrichment of multiple genera and network-style degradation.

In summary, the vast majority of polysaccharides demonstrating clear efficacy for UC possess a composite structural pattern of “β-type backbone + acidic/high-mannose components + multiple branches + medium molecular weight (10–200 kDa)”. This configuration can simultaneously satisfy the substrate preferences of multiple classes of beneficial bacteria, driving more robust microbial cross-feeding and metabolic synergy, thereby exerting integrated regulatory effects across multiple aspects, including anti-inflammation, barrier repair, and oxidative stress mitigation.

## 4. Remodeling of Microbial Communities and Production of Functional Metabolites in UC

Natural polysaccharides, as a class of complex carbohydrates not degraded by the host’s upper digestive tract enzyme systems, exert their core therapeutic mechanism in UC by acting as targeted prebiotics that precisely remodel the imbalanced gut microbiota [75]. Data integrated in this study indicate that polysaccharides with different structural characteristics can drive specific, predictable taxonomic shifts, thereby restoring the homeostasis and ecological functions of the gut microbiome. This transformation is not only reflected in abundance changes at the phylum and genus levels but, more profoundly, promotes the restoration of microbial community functions, particularly the capacity to produce SCFAs.

### 4.1. Targeted Enrichment and Suppression of Functional Microbiota at the Phylum Level

In the pathological state of UC, the gut microbiota typically exhibits an imbalance in the Firmicutes to Bacteroidetes ratio (F/B ratio) and an abnormal expansion of Proteobacteria [76]. The polysaccharide intervention studies included in our analysis show that polysaccharides can effectively correct this imbalance, but the direction of modulation is significantly dependent on the species source and chemical structure, which is mainly reflected in the following three aspects.

The first aspect is the promotion of Bacteroidetes proliferation. Our evidence mapping shows that the vast majority of pectin-type polysaccharides rich in acidic monosaccharides (especially galacturonic acid), such as those from *Lycium barbarum* (galacturonic acid content 24.67%), *Citrus medica* (91.47%), *Diospyros lotus* (75.49%), *Cyclocarya paliurus* (14.2%), and *Porphyra haitanensis* (rich in sulfated galactose), significantly upregulated the relative abundance of Bacteroidetes in DSS-induced UC mouse models. Additionally, polysaccharides from *Thesium chinense* (mainly neutral sugars), *Asimina triloba* (containing a proportion of acidic sugars), and *Sagittaria sagittifolia* (mainly neutral glucan) also showed the same trend. Bacteroidetes are the main force for degrading complex dietary fibers and polysaccharides. Their proliferation indicates that these exogenous polysaccharides are effectively utilized by the host’s symbiotic microbiota. This process is often accompanied by an increased production of SCFAs like propionate and acetate, which is directly correlated with the observed effects in studies, such as intestinal barrier repair (upregulated tight junction protein expression), elevated levels of anti-inflammatory factors (e.g., IL-10), and reduced levels of pro-inflammatory factors (e.g., IL-6, TNF-α).

The second aspect is the promotion of Firmicutes proliferation. Our evidence mapping showed that polysaccharides based on neutral glucans and some mannans tend to preferentially enrich Firmicutes. For example, glucose-rich polysaccharides from Flower mushroom (β-glucan, 100% glucose), *Zingiber officinale* Roscoe (85.09% glucose), and *Gastrodia elata* Blume (>99% glucose), as well as mannose-rich polysaccharides from *Aloe* (84.2% mannose) and *Bletilla striata* (70.68% mannose), all significantly upregulated Firmicutes abundance after intervention. Polysaccharides from *Grifola frondosa* (86.9% glucose), *Tremella aurantialba*, and *Laminaria japonica* also showed similar effects. Firmicutes contain a large number of butyrate-producing genera [77]. Butyrate is the primary energy source for colonic epithelial cells and is crucial for maintaining epithelial barrier integrity, regulating immune balance, and exerting anti-inflammatory effects [78]. These research findings support the mechanism whereby such polysaccharides improve colonic energy metabolism and reduce inflammation and tissue damage by promoting the growth of butyrate-producing bacteria.

The third aspect is the suppression of Proteobacteria expansion. Multiple clinical and preclinical studies indicate that the overgrowth of Proteobacteria (especially the γ-proteobacteria class) is a core hallmark of dysbiosis in UC, and its abundance is positively correlated with the severity of intestinal inflammation [79,80]. Our analysis shows that both the aforementioned acidic and neutral polysaccharides generally possess the ability to significantly downregulate Proteobacteria abundance in UC models. For instance, interventions with polysaccharides from *Hericium erinaceus*, Barley bran, *Asimina triloba*, *Astragalus membranaceus*, Flower mushroom, *Zingiber officinale* Roscoe, *Cyclocarya paliurus*, *Phyllostachys edulis*, etc., all observed a reduction in Proteobacteria abundance. This effect may stem from polysaccharides promoting competitive occupation by beneficial bacteria, their fermentation products altering the gut microenvironment (e.g., lowering pH), or the direct inhibition of pathogen growth, thereby effectively reducing the colonization and expansion of potential pathogens (e.g., *Escherichia*–*Shigella*, *Helicobacter*) and alleviating intestinal inflammation.

In summary, plant and fungal polysaccharides from different sources and structures remodel the gut microecological balance in the UC state by specifically modulating the relative abundances of three key phyla—Firmicutes, Bacteroidetes, and Proteobacteria—thereby synergistically exerting therapeutic effects including anti-inflammation, anti-oxidation, intestinal barrier repair, and colon tissue protection. The direction of modulation is closely related to structural features of the polysaccharides, such as monosaccharide composition, backbone linkage patterns, and charge properties.

### 4.2. Targeted Enrichment and Suppression of Functional Microbiota at the Genus Level

The finer regulation of microbiota by polysaccharides is also manifested at the genus level, where their structural specificity guides the targeted enrichment of specific functional groups.

First, regarding enrichment of core SCFA-producing genera, various active polysaccharides with different structures demonstrate the ability to upregulate genera closely related to SCFA (especially butyrate, acetate, and propionate) production. Butyrate, as the main energy source for colonic epithelial cells, is crucial for maintaining the intestinal barrier [81]. Polysaccharides with complex structures, often containing β-(1→3) or β-(1→4) glucose/mannose backbones and branches (e.g., from *Grifola frondosa*, Flower mushroom), can specifically enrich key butyrate producers, such as *Lachnospiraceae* members like *Lachnospiraceae*_NK4A136_group and Roseburia, as well as members of Ruminococcaceae, which also significantly increased after interventions with *Lycium barbarum* and *Astragalus membranaceus* polysaccharides. For the main producers of acetate and propionate, polysaccharide regulation is more targeted. Pectin-like or heteropolysaccharides rich in neutral sugars like arabinose and galactose (e.g., from *Dioscorea opposita* Maxim, *Porphyra haitanensis*) can significantly upregulate the abundance of the genus *Bacteroides*; whereas polysaccharides rich in fructose or specific galactose structures (e.g., the fructan from *Thesium chinense*) tend to enrich the genus *Prevotella*. Furthermore, polysaccharides from *Asimina triloba* and *Tremella aurantialba* can also promote the growth of the genus *Bifidobacterium*, whose metabolites, acetate and lactate, can further promote the proliferation of butyrate-producing bacteria via cross-feeding.

Second, regarding promoting genera with barrier-protective functions, multiple polysaccharides can directionally enrich probiotics that directly or indirectly enhance intestinal barrier integrity. The most frequently observed is *Akkermansia* muciniphila, which utilizes mucin as a carbon source, stimulates mucus layer thickening, and enhances tight junctions [82]. Polysaccharides rich in mannose or with specific glucan/mannan structures that mimic mucin (e.g., from *Tremella aurantialba*, *Aloe*), as well as polysaccharides from sources like *Zingiber officinale* Roscoe and *Citrus medica*, can effectively upregulate its abundance. The genus *Lactobacillus*, as a classic probiotic, shows extremely widespread enrichment across numerous polysaccharide interventions (e.g., *Asimina triloba*, *Grifola frondosa*, *Porphyra haitanensis*, *Bletilla striata*, and *Diospyros lotus* polysaccharides), which is related to its preference for utilizing monosaccharides like glucose and mannose, as well as some polysaccharide degradation products. *Lactobacillus* not only inhibits pathogens by producing lactic acid to lower luminal pH, but its metabolites can also serve as substrates converted by other beneficial bacteria into barrier-protective butyrate [83].

Third, regarding suppression of pro-inflammatory and potential pathogenic genera, polysaccharide intervention can effectively inhibit pro-inflammatory and potential pathogenic bacteria, which are often abnormally proliferated in UC. This inhibitory effect may be achieved indirectly through competition for nutrients, promotion of inhibitory metabolite production by beneficial bacteria, or direct modulation of host immunity. The abundance of *Escherichia*–*Shigella* significantly decreased after intervention with arabinoxylans (e.g., Barley bran), pectin (e.g., *Asimina triloba*), or specific heteropolysaccharides (e.g., *Phyllostachys edulis*). The genus *Helicobacter* is sensitive to polysaccharides from *Thesium chinense*, Barley bran, *Phyllostachys edulis*, etc. *Desulfovibrio*, a potentially harmful bacterium that produces hydrogen sulfide, also showed significant downregulation in abundance after treatment with *Thesium chinense* and *Ishige okamurae* polysaccharides. It is noteworthy that the reduction in these pro-inflammatory genera is highly synchronized in timing and degree with the observed decreases in colonic inflammatory factors (e.g., IL-6, TNF-α), oxidative stress markers, and improvements in histopathology in the models, suggesting that remodeling the microbiota structure and inhibiting the overgrowth of harmful bacteria is one of the key mechanisms by which polysaccharides exert their anti-inflammatory and mucosal repair effects. Additionally, *Astragalus membranaceus* and Flower mushroom polysaccharides can specifically downregulate other genera associated with UC inflammatory states (e.g., *Parasutterella*, *Romboutsia*), further refining the regulation of microecological balance.

### 4.3. Polysaccharide-Driven Modulation of Gut Microbiota Is Primarily Mediated by CAZymes

The underlying mechanism by which natural polysaccharides drive specific taxonomic shifts in the gut microbiota lies at the core of their complex chemical structures, which serve as selective metabolic codes. Through a sequential interaction involving structure as well as enzyme and microbial communities, they accomplish precise nutritional support and habitat modulation for particular functional groups of microorganisms. Please see Table 2 for specifics. According to evidence mapping, this process follows three key mechanisms:

First, glycosidic bond type and backbone conformation determine the specificity of Primary Degraders. Microbial utilization of polysaccharides begins with hydrolysis reactions catalyzed by CAZymes [84]. Different CAZyme families exhibit high specificity for glycosidic bond types (e.g., α- or β-linkages), sugar ring conformations (pyranose or furanose), and linkage positions (e.g., 1→3, 1→4, 1→6) [85]. For example, β-glucans (e.g., (1→3)-β-glucan from Flower mushroom) are primarily degraded by enzymes from glycoside hydrolase (GH) 16, GH17, and GH55 families, and these enzyme genes are enriched in Firmicutes genera such as *Lachnospiraceae*_NK4A136_group and *Clostridia*, explaining why such polysaccharides specifically drive Firmicutes proliferation. Conversely, pectin-type polysaccharides (e.g., *Citrus medica*) rich in α-1,4-linked galacturonan require GH28, polysaccharide lyase(PL), and carbohydrate esterase(CE) 8 family enzymes for depolymerization. These enzyme systems are core metabolic equipment for the Bacteroidetes phylum (especially the genus *Bacteroides*), thus directionally enriching Bacteroidetes. Therefore, the primary structure (glycosidic bond type) of a polysaccharide determines that it can only be recognized and utilized by Primary Degraders carrying the corresponding CAZymes. This is the first key mechanism driving taxonomic shifts.

Second, side-chain modifications and branching structures regulate microbial collaboration networks and cross-feeding. Many active polysaccharides are not simple linear structures but possess abundant side chains [86]. These side chains constitute a secondary metabolic barrier, often requiring additional, specific debranching or modifying enzymes to remove them first before exposing the backbone for further degradation [21]. For example, the arabinogalactan side chains of *Phyllostachys edulis* polysaccharide are rich in arabinose and galactose, requiring GH43, GH51, and GH36 enzymes from genera like *Prevotella* and *Bifidobacterium* for preprocessing. This complexity fosters “division of labor and cooperation” among microbes. Initial degraders may be responsible for debranching or cleaving the backbone, producing oligosaccharides or monosaccharides. These intermediate products are then utilized by secondary utilizers (e.g., butyrate-producing Roseburia, Faecalibacterium) that lack a complete degradative enzyme system but possess specific uptake and fermentation systems—a process also defined as cross-feeding [87]. For instance, after initial degradation of *Asimina triloba* arabinoglucan by *Lactobacillus*, the produced arabinose may promote *Bifidobacterium* growth, whose metabolic product, acetate, can then serve as a substrate for *Lachnospiraceae* to convert into butyrate. Therefore, the side-chain complexity of polysaccharides is not a barrier to utilization but, by constructing intricate metabolic steps, shapes an orderly, functionally coupled microbial collaboration network.

Third, charge properties and physicochemical characteristics shape niche selection pressure. The charge properties and solubility (all polysaccharides discussed here are water-soluble) of polysaccharides directly influence their physical form, interaction with the intestinal mucosa, and ecological selection pressure on microorganisms [88]. Acidic polysaccharides, due to their negative charge, more easily interact with positively charged components in the intestinal mucus layer, forming mucus–polysaccharide complexes, thereby enriching niches closer to the epithelium [89]. This can not only physically strengthen the mucus barrier but also provides a dedicated “micro-carbon source” for probiotics that prefer acidic sugars (e.g., some *Lactobacillus*, *Akkermansia*) colonizing this area [90]. For example, highly methoxylated pectin from *Citrus medica* might be more readily utilized by *Muribaculaceae* carrying specific esterases (CE8). The sulfate groups of sulfated polysaccharides from seaweeds have unique bioactivity, and the distribution of some sulfatases (e.g., GH107) is genus-specific (e.g., *Bacteroides*), constituting another layer of selection. Furthermore, SCFAs produced from polysaccharide fermentation rapidly lower the local pH. This acidified microenvironment selectively inhibits potential pathogens preferring neutral environments (e.g., many members of *Escherichia*–*Shigella*, *Proteobacteria*) while promoting the growth of acid-tolerant beneficial bacteria (e.g., lactic acid bacteria and butyrate producers) [91]. This environmental screening driven by polysaccharide metabolites is a key subsequent mechanism for inhibiting harmful bacteria like Proteobacteria and consolidating the ecological advantage of beneficial bacteria.

### 4.4. Functional Restoration of the Microbial Community Centered on SCFA Production

Polysaccharide-driven taxonomic shifts ultimately serve to restore the ecological functions of the microbial community. The most critical evidence is that in nearly all studies, the enrichment of beneficial bacteria (such as *Lachnospiraceae*, *Ruminococcaceae*, and *Bacteroides*) coincides with phenotypic improvements in the host, including restored colon length, upregulation of tight junction protein expression (ZO-1, Occludin, and Claudin-1), and alleviation of inflammation. This indicates that polysaccharides activate the metabolic activity of these SCFA-producing bacteria by providing them with exclusive substrates. SCFAs (particularly butyrate and propionate) have been shown to provide energy for intestinal epithelial cells and promote barrier repair [92]; they exert anti-inflammatory and immunomodulatory effects by activating G protein-coupled receptors (GPR41/43, GPR109a) and inhibiting histone deacetylases [93], and they regulate the luminal pH to inhibit pathogen growth [94]. Therefore, the essence of polysaccharide intervention lies in the precise feeding and activation of the host’s inherent SCFA-producing microbiota through the substrate specificity encoded by their chemical structures, thereby restoring the microbial metabolic functional output crucial for host health.

## 5. The Multi-Target Therapeutic Effects of Polysaccharides on Pathological Improvement in UC

**Figure 2:**
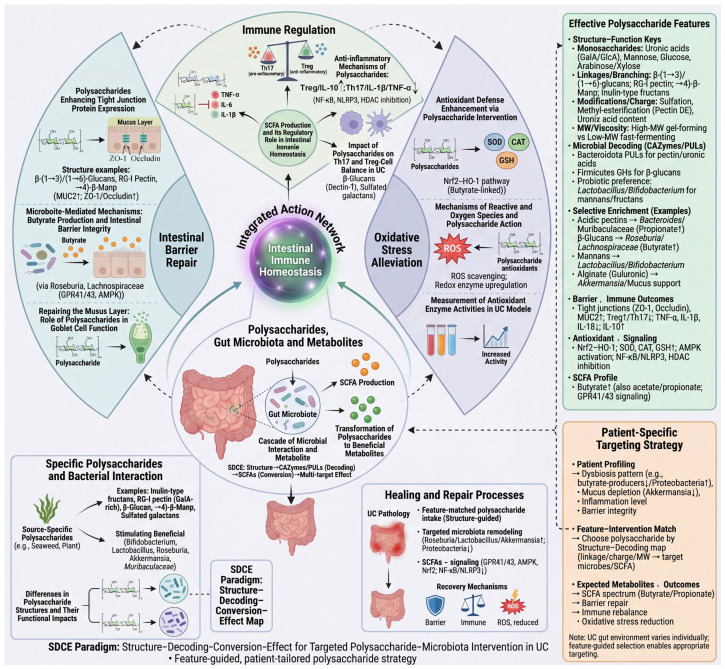
Integrated network of dietary polysaccharides sustaining intestinal immune homeostasis. Abbreviations: UC: ulcerative colitis; SCFAs: short-chain fatty acids; ROS: reactive oxygen species; RNS: reactive nitrogen species; Nrf2: nuclear factor erythroid 2–related factor 2; NF-κB: nuclear factor kappa B; NLRP3: NOD-like receptor family pyrin domain-containing 3; STAT: signal transducer and activator of transcription 3; Treg: regulatory T cell; Th17: T helper 17 cell; Th1: T helper 1 cell; IL-10: interleukin-10; IL-1β: interleukin-1 beta; IL-17: interleukin-17; IL-19: interleukin-19; IFN-γ: interferon-gamma; ZO-1: zonula occludens-1; MUC2: mucin 2; MUC5AC: mucin 5AC; AMPK: AMP-activated protein kinase; MAPK: mitogen-activated protein kinase; GSH: reduced glutathione; CAT: catalase; SOD: superoxide dismutase; HO-1: heme oxygenase-1; Dectin-1: dendritic cell-associated *C*-type lectin-1; TLR: Toll-like receptor; CAZymes: carbohydrate-active enzymes; and RG-I: rhamnogalacturonan I.

Following the process of Structure–Decoding–Conversion–Effect, we arrive at the stage where natural polysaccharides drive the effect exertion of gut microbiota. Through multi-level interventions (Figure 2), they ultimately alleviate the three core pathological changes in UC: intestinal barrier disruption, immune-inflammatory imbalance, and oxidative stress [95].

### 5.1. Repairing the Intestinal Mucosal Physical and Chemical Barriers

Loss of intestinal barrier function is a key link in the occurrence and development of UC [96]. Polysaccharides reinforce the barrier through microbiota-mediated mechanisms at multiple levels. In terms of enhancing the expression of tight junction proteins, 20 kinds of polysaccharides from sources such as Barley bran, *Asimina triloba*, *Sagittaria sagittifolia* L., and *Ishige okamurae* can significantly upregulate the expression of tight junction proteins like ZO-1, Occludin, and Claudin-1. This effect is primarily attributed to their promotion of butyrate-producing bacteria (e.g., *Lachnospiraceae*, *Faecalibacterium prausnitzii*) proliferation. The produced butyrate can directly act on intestinal epithelial cells, promoting the assembly and expression of tight junction proteins by activating signaling pathways such as AMPK [97]. The mucus layer is the first chemical barrier separating gut microbiota from the epithelium [98]. Regarding promoting mucus layer repair, *Laminaria japonica* can directly increase O-glycan synthesis in the mucus layer. Meanwhile, upregulating *Akkermansia* (e.g., via *Tremella aurantialba* polysaccharide) or Lactobacilli can optimize mucus metabolism balance. On the one hand, this stimulates goblet cells to secrete mucin MUC2 (e.g., Flower mushroom, *Bletilla striata* polysaccharides); on the other hand, it prevents excessive mucus degradation, thereby increasing mucus layer thickness. In maintaining goblet cell numbers and function, polysaccharides from sources like *Dioscorea opposita* Maxim, *Asimina triloba*, and *Grifola frondosa* can effectively alleviate UC-induced goblet cell loss. This is closely related to SCFAs (especially butyrate) providing energy for goblet cells and regulating their differentiation and maturation [99].

### 5.2. Regulating Intestinal Immune Homeostasis and Suppressing Excessive Inflammation

Polysaccharides regulate the intestinal immune system through microbial metabolites and the microbial community structure itself [80]. The characteristic excessive activation of Th17 cells and functional suppression of Treg cells in UC can be reversed by various polysaccharides (e.g., Flower mushroom). SCFAs, particularly butyrate and propionate, are key molecules inducing the differentiation and function of intestinal mucosal Treg cells [100]. They can promote the production of the anti-inflammatory cytokine IL-10 (e.g., *Thesium chinense*, *Asimina triloba* polysaccharides) through histone deacetylase (HDAC) inhibition and GPR signaling, while simultaneously inhibiting the secretion of pro-inflammatory cytokines IL-6, IL-17, and TNF-α (reported in all studies). *Gastrodia elata* Blume polysaccharide can also inhibit the activation of the NLRP3 inflammasome, a crucial upstream link controlling the maturation and release of IL-1β. Inflammation is another characteristic manifestation of UC [96]. SCFAs produced by polysaccharide fermentation can inhibit the activation of signaling pathways like NF-κB, thereby downregulating the expression of various pro-inflammatory mediators at the transcriptional level [101]. Furthermore, certain polysaccharides or their degraded fragments might be recognized by pattern recognition receptors on the surface of dendritic cells or macrophages, directly inducing anti-inflammatory immune responses [16]. Additionally, polysaccharides can alleviate abnormal immune cell infiltration under UC conditions [102]. For example, *Porphyra haitanensis* polysaccharide can reduce pathological infiltration of CD4^+^ and CD8^+^ T cells in colonic tissue, indicating its ability to systemically alleviate local immune cell recruitment and activation in the intestine.

### 5.3. Alleviating Oxidative Stress and Improving Systemic Metabolism

The reactive oxygen species (ROS) burst accompanying UC leads to oxidative damage [103]. Polysaccharide intervention can systemically enhance antioxidant defense capacity. Polysaccharides from sources like *Dioscorea opposita* Maxim, *Thesium chinense*, and *Cyclocarya paliurus* can significantly increase the activity of superoxide dismutase (SOD), catalase (CAT), and glutathione peroxidase (GSH-Px), while reducing the levels of lipid peroxidation products such as malondialdehyde (MDA). This effect may partly originate from SCFAs activating the expression of host antioxidant enzyme genes via the Nrf2 signaling pathway [104]. On the other hand, myeloperoxidase (MPO) is a marker of neutrophil activation. Reducing MPO activity (e.g., via *Thesium chinense*, *Cyclocarya paliurus* polysaccharides) directly reflects the alleviation of intestinal inflammation and reduction in neutrophil infiltration.

### 5.4. Constructing an Integrated “Structure–Decoding–Conversion–Effect” Action Network

In summary, the alleviation of UC by natural polysaccharides is an integrated cascade process, which can be summarized as a linear logic network: “Structure (Specific Substrate) → Recognition (Taxonomic and Functional Remodeling) → Transformation (Key Metabolite Production) → Effect (Multi-Target Host Response)”. Specifically, polysaccharides with specific monosaccharide composition, molecular weight, and glycan chain structure are preferentially recognized and fermented by specific bacterial taxa within the gut microbiota that possess corresponding CAZymes. This fermentation directionally enriches beneficial functional microbiota, with SCFA production at its core, and inhibits pro-inflammatory pathogenic bacteria. The proliferated beneficial bacteria transform the polysaccharides into a series of beneficial metabolites represented by SCFAs. These metabolites, acting as key signaling molecules and energy sources, directly affect intestinal epithelial cells and immune cells. By activating (e.g., Nrf2) or inhibiting (e.g., NF-κB) specific host signaling pathways, they ultimately synergistically achieve the comprehensive therapeutic goals of repairing the intestinal barrier, suppressing excessive inflammation, and alleviating oxidative stress. Therefore, the essence of natural polysaccharides may be a type of “prebiotic precursor” [105]. Their chemical structure determines the manner in which they regulate microbial ecology, and subsequently, through microbial metabolic transformation, they achieve therapeutic and reparative effects on the UC pathological state and the gut microecological environment.

## 6. Conclusions and Perspectives

UC is a chronic inflammatory bowel disease with a complex etiology and recurrent course, whose pathogenesis is closely related to the imbalance of intestinal microbiota homeostasis. Traditional drug therapies primarily focus on symptom control and often struggle to fundamentally restore the structure of the intestinal microecology and achieve long-term remission. Due to their unique “indigestibility” and precise potential for microbiota modulation, natural polysaccharides are increasingly becoming a core research focus for novel UC treatment strategies based on microecological intervention. This review systematically examines relevant research from the past decade, with a focus on analyzing how natural polysaccharides with different structural features drive the directional remodeling of gut microbiota through a “Structure–Decoding–Conversion–Effect” paradigm, ultimately synergistically alleviating the pathological progression of UC.

The analysis presented indicates that natural polysaccharides are not merely simple fermentable substrates, but a class of ecological modulators with defined structural coding, whose bioactivity is highly dependent on their structural features: the vast majority of polysaccharides demonstrating clear therapeutic efficacy against UC share a composite structural pattern characterized by a “β-type backbone + acidic/high-mannose components + multi-branching + medium molecular weight (10–200 kDa)”. These structural parameters collectively constitute the structural coding of polysaccharides, enabling them to be recognized and preferentially utilized by specific CAZyme systems within gut microorganisms. This facilitates the targeted enrichment of specific functional bacterial groups, such as *Roseburia*, *Lactobacillus*, and *Akkermansia*, alongside the competitive inhibition of pro-inflammatory genera like *Escherichia*–*Shigella* and *Helicobacter*. This structure-dependent microbiota remodeling is the fundamental starting point for the therapeutic action of natural polysaccharides.

The gut microbiota possesses not only polysaccharide recognition functions but also crucial transformation capabilities. The profound significance of polysaccharide-driven changes in microbial community structure lies in restoring and enhancing the functional output of the microbial community, with the production of SCFAs being the most central metabolic hub. Directionally enriched butyrate-producing bacteria like *Lachnospiraceae* and propionate-producing bacteria like *Bacteroides* transform the structurally specific polysaccharides into SCFAs such as butyrate and propionate. These metabolites act as key signaling molecules and energy substrates connecting microbial activity with host physiology. Through the aforementioned cascade, natural polysaccharides ultimately exhibit multi-target, multi-level integrated therapeutic effects at the host level: repairing the intestinal barrier, modulating immune inflammation, and alleviating oxidative stress.

Despite the great potential shown by natural polysaccharides in the microecological treatment of UC, translating them into precise clinical intervention strategies still faces numerous challenges and opportunities. Firstly, the structure–activity relationship remains insufficiently deciphered. Current understanding of polysaccharide-driven microbiota relationships largely remains at the correlational level. Future research needs to integrate synthetic biology, molecular simulation, and more advanced omics technologies to elucidate at the molecular level how specific glycosidic bonds or side chains are recognized by specific CAZymes, and to accurately predict their downstream microbial succession and metabolic output. Secondly, the vast majority of existing studies are based on rodent models, whose gut microbiota differs from that of humans. There is a need for high-quality, rigorously designed clinical studies to verify the safety, efficacy, and optimal dosage of specific structured polysaccharides in UC patients, and to establish personalized response prediction models based on patients’ baseline microbiota characteristics. In addition, due to the high heterogeneity between models and outcomes (including animal species, DSS induction protocols, intervention doses/durations, indicator systems, etc.; for details, see Table S1), this review remains primarily narrative in its synthesis. High-quality meta-analyses are still needed in the future to further decode the complex interactions between polysaccharides and gut microbiota in UC interventions.

In summary, natural polysaccharides, through the substrate specificity encoded in their chemical structures, precisely drive the structural and functional remodeling of gut microbiota. Subsequently, through microbial metabolites, with SCFAs at the core, they repair the intestinal barrier via a multi-target approach, suppress excessive inflammation, and alleviate oxidative stress, thereby generating comprehensive therapeutic effects against UC. This reveals a clear axis of action from “chemical structure” to “ecological function” and finally to “host health”. Future research should be dedicated to deepening the mechanistic understanding of this “Structure–Decoding–Conversion–Effect” network and promoting the translation of precision microecological intervention strategies based on polysaccharide structure from the laboratory to the clinic, providing safer and more effective microecological solutions for the prevention and treatment of complex diseases like UC.

## Figures and Tables

**Table 2:** CAZyme-mediated microbial polysaccharide-driven mode.

Structural Category	Source	Glycan Structural Features	Relevant CAZymes	Corresponding Bacterial Genera (Original Study)
I. β-Glucans				
(1→3)-β-Glucan	*Flower Mushroom* [42]	(1→3)-β-D-Glcp	GH16, GH17, GH55, GH81, GH128	*Lachnospiraceae*_NK4A136_group, *Clostridia*, *Odoribacter*
(1→3,1→4)-β-Glucan	*Hericium erinaceus* [36]	α/β-D-Glc(1→3)	GH16, GH5, GH12	*Ruminococcaceae*, *Lachnospiraceae*, *Akkermansia*, *Allobaculum*
II. α-Glucans				
Highly Branched α-Glucan	*Grifola frondosa* [43] (side chain)	Side chain: Glcp-(1→; backbone contains branching points	GH13, GH5, GH35, GH38	*Lactobacillus*, *Lachnospiraceae*, *Roseburia*
Highly Branched α-Glucan	*Zingiber officinale* Roscoe [50]	Backbone: →4,6)-β-Glcp-1→ and →3,6)-α-Galp-1	GH13, GH5, GH36, GH43	*Muribaculaceae*
Highly Branched α-Glucan	*Sagittaria sagittifolia* L. [54]	Backbone: →4)-Glcp-(1→,→6)-Glcp-(1→	GH13, GH15, GH35, GH38	*Lactobacillus*, *Candidatus_Saccharimonas*, *Nitrosospira*, *Dialister*
Branched α-Glucan	*Gastrodia elata* Blume [51]	→4)-α-D-Glcp(1→,containing →4,6) branching points	GH13, GH57	-
III. Glucomannans				
Glucose–Mannose Copolymer	*Dioscorea opposita* Maxim [34]	Backbone: →4)-α-D-Glc,→4)-β-D-Man	GH13, GH5, GH26	*Bacteroides*, *Prevotella*, *Phascolarctobacterium*
Glucose–Mannose Copolymer (Pectin-like Backbone)	*Grifola frondosa* [43] (backbone)	Backbone: →4)-Glcp-(1→,→4)-Galp-(1→,→3,6)-Manp-(1→	GH13, GH16, GH5	*Lactobacillus*, *Lachnospiraceae*, *Roseburia*
IV. Arabinoglucans/Arabinogalactans				
Arabinoglucan	*Asimina triloba* [38]	Backbone:→4)-α-D-Glcp-(1→; side chains rich in arabinose (31.9%)	GH13, GH43, GH51	*Lactobacillus*, *Muribaculum*, *Bifidobacterium*, *Turicobacter*
Arabinogalactan	*Phyllostachys edulis* [56]	Backbone: β-(1→4)-Glcp; side chains rich in galactose (26.8%) and arabinose	GH5, GH6, GH35, GH36, GH43	*Prevotella*, *Alistipes*, *Anaerostipes*, *Odoribacter*, *Bifidobacterium*, *Butyricimonas*, *Lactobacillus*
V. Mannans				
(1→4)-β-Mannan	*Aloe* [48]	Backbone: →4)-β-Manp-(1→	GH5, GH26, GH113	*Akkermansia*, *Turicobacter*
(1→4)-β-Mannan	*Bletilla striata* [55]	Backbone: →4)-β-D-Manp-(1→),→4)-β-D-Glcp-(1→	GH5, GH26, GH113, GH9	*Lactobacillus*, *Muribaculaceae*
(1→3)-β-Mannan	*Tremella aurantialba* [44]	Backbone: →3)-Manp-(1→	GH39, GH134	*Akkermansia*, *Adlercreutzia*, *Lactobacillus*, *Bifidobacterium*
Galactomannan	*Areca catechu* L. [53]	β-1,4-Mannan backbone, side chains with galactose, arabinose	GH5, GH26, GH113; GH27, GH36; GH43, GH51	*Ligilactobacillus*, *Dubosiella*, *Lactobacillus*, *Ruminococcus*
VI. Xylans				
Arabinoxylan	Barley bran [37]	Backbone: β-1,4-Xylan; side chains: arabinose	GH10, GH11, GH30; GH43, GH51, GH54, GH62	*Ligilactobacillus*, *Lachnospiraceae_NK4A136_group*
VII. Fructans				
β-(2→6)-Fructan (Levan)	*Thesium chinense* Turcz [35]	→1)-β-D-Fruf-(2→,→1,6)-β-D-Fruf-(2→	GH32, GH91	*Ruminococcus*, *Ligilactobacillus*, *Alloprevotella*, *Clostridia*_UCG-014, *Bifidobacterium*
VIII. Pectic Polysaccharides				
HG	*Citrus medica* [47]	→4)-α-D-GalAp-6-O-CH_3_-(1→	GH28, PL1, PL2, PL3, PL9, PL10, CE8	*Muribaculaceae*, *Clostridia*, *Lachnospiraceae*, *Alistipes*, *Butyricimonas*
HG	*Diospyros lotus* L. [46]	→4)-α-GalpA-(1→	GH28, PL1, PL2, PL3, PL9, PL10, CE8	*Lachnospiraceae*, *Lactobacillaceae*
RG-I	*Lycium barbarum* L. [39]	[→4)-α-D-GalAp-(1→2)-α-L-Rhap-(1→]n, side chains of arabinan/galactan	GH28, PL1, PL2, PL3, PL9, PL10, CE8, GH106; GH43, GH51, GH35, GH36	*Muribaculaceae*, *Lactobacillus*, *Lachnospiraceae_NK4A136_group*
RG-I	*Cyclocarya paliurus* [49]	→4)-α-D-GalpA-(1→,→2)-α-L-Rhap-(1→4)-α-D-GalpA-(1→	GH28, PL1, PL2, PL3, PL9, PL10, CE8, GH106; GH43, GH51, GH35, GH36	*Alistipes*, *Sphingomonas*, *Lactobacillus*, *Coprococcus*
IX. Galactans				
β-(1→6)-Galactan	*Astragalus membranaceus* [41]	Backbone: →6)-β-Galp-(1→4)-β-Galp-(1→	GH35, GH2	*Muribaculaceae*, *Lachnospiraceae*, *Rikenellaceae*, *Ruminococcaceae*, *Prevotellaceae*
β-(1→6)-Galactan (Pectin-like)	*Dioscorea opposita* Maxim [49]	Backbone: →6)-β-D-Galp-(1→	GH35, GH2	*Bacteroides*, *Mucispirillum*, *Lactobacillus*, *Coprococcus*
X. Marine Polysaccharides				
Agarose/Carrageenan	*Porphyra haitanensis* [40]	→3)-β-D-Galp-(1→4)-α-L-Galp-6S-(1→	GH16, GH86, GH117, GH82, GH127	*Bacteroides*, *Muribaculum*, *Lactobacillus*
Alginate (Polyguluronate)	*Laminaria japonica* [45]	→4)-α-L-GulAp-(1→	PL5, PL6, PL7, PL14, PL15, PL17, PL18	*Lactobacillus*, *Muribaculaceae*, *Prevotellaceae*
Fucoidan	*Ishige okamurae* [52]	→3)-α-L-Fucp-(1→,→4)-α-L-Fucp-(1→	GH107, GH168, CE4	*Muribaculaceae*, *Lachnospiraceae*, *Rikenella*

Glc: glucose; Gal: galactose; Man: mannose; Fuc: fucose; Xyl: xylose; Ara: arabinose; and Fru: fructose. GalA (GalAp): D-galacturonic acid; GlcA (GlcAp): D-glucuronic acid; GulA (GulAp): L-guluronic acid; and ManA: D-mannuronic acid. HG: homogalacturonan; RG-I: rhamnogalacturonan-I. GH: glycoside hydrolase family; PL: polysaccharide lyase family; and CE: carbohydrate esterase family. Numerals (e.g., GH16, PL9, and CE8) denote CAZy families. →: direction of glycan chain elongation.

## Data Availability

The author will provide all original data upon request.

## Supplementary Materials

The following supporting information can be downloaded at: https://www.mdpi.com/article/10.3390/nu18081297/s1, Table S1: Characteristics of intervention protocols for polysaccharides from different sources in animal models of ulcerative colitis.

## Abbreviations

The following abbreviations are used in this manuscript:

AMPK	AMP-activated protein kinase
CAZymes	Carbohydrate-active enzymes
CAT	Catalase
DAI	Disease activity index
DSS	Dextran sulfate sodium
ET	Endotoxin
F/B ratio	Firmicutes/Bacteroidetes ratio
GH	Glycoside hydrolase
GPR	G protein-coupled receptor
GSH-Px	Glutathione peroxidase
HDAC	Histone deacetylase
HG	Homogalacturonan
IL	Interleukin
LBP	Lipopolysaccharide-binding protein
MAPK	Mitogen-activated protein kinase
MCP-1	Monocyte chemoattractant protein-1
MDA	Malondialdehyde
MPO	Myeloperoxidase
MUC2	Mucin 2
NF-κB	Nuclear factor kappa-B
NLRP3	NOD-like receptor family pyrin domain-containing 3
Nrf2	Nuclear factor erythroid 2-related factor 2
PCNA	Proliferating cell nuclear antigen
PL	Polysaccharide lyase
PULs	Polysaccharide utilization loci
RG-I	Rhamnogalacturonan I
ROS	Reactive oxygen species
SCFAs	Short-chain fatty acids
SOD	Superoxide dismutase
STAT3	Signal transducer and activator of transcription 3
Th17	T helper 17 cells
TNF-α	Tumor necrosis factor-alpha
Tregs	Regulatory T cells
UC	Ulcerative colitis
ZO-1	Zonula occludens-1
FOSs	Fructooligosaccharides

## References

[1] Boland K., Bedrani L., Turpin W., Kabakchiev B., Stempak J., Borowski K., Nguyen G., Steinhart A.H., Smith M.I., Croitoru K. (2021). Persistent Diarrhea in Patients with Crohn’s Disease After Mucosal Healing Is Associated with Lower Diversity of the Intestinal Microbiome and Increased Dysbiosis. Clin. Gastroenterol. Hepatol..

[2] Le Berre C., Honap S., Peyrin-Biroulet L. (2023). Ulcerative colitis. Lancet.

[3] Kobayashi T., Siegmund B., Le Berre C., Wei S.C., Ferrante M., Shen B., Bernstein C.N., Danese S., Peyrin-Biroulet L., Hibi T. (2020). Ulcerative colitis. Nat. Rev. Dis. Primers.

[4] van de Guchte M., Mondot S., Doré J. (2021). Dynamic Properties of the Intestinal Ecosystem Call for Combination Therapies, Targeting Inflammation and Microbiota, in Ulcerative Colitis. Gastroenterology.

[5] Franzosa E.A., Sirota-Madi A., Avila-Pacheco J., Fornelos N., Haiser H.J., Reinker S., Vatanen T., Hall A.B., Mallick H., McIver L.J. (2019). Gut microbiome structure and metabolic activity in inflammatory bowel disease. Nat. Microbiol..

[6] Nishino K., Nishida A., Inoue R., Kawada Y., Ohno M., Sakai S., Inatomi O., Bamba S., Sugimoto M., Kawahara M. (2018). Analysis of endoscopic brush samples identified mucosa-associated dysbiosis in inflammatory bowel disease. J. Gastroenterol..

[7] Han A., Yang M., Chen B., Cao G., Xu J., Meng T., Liu Y., Wang Z., Zhou Y., Xu N. (2024). Microbiome and its relevance to indigenous inflammatory bowel diseases in China. Gene.

[8] Kałużna A., Olczyk P., Komosińska-Vassev K. (2022). The Role of Innate and Adaptive Immune Cells in the Pathogenesis and Development of the Inflammatory Response in Ulcerative Colitis. J. Clin. Med..

[9] Wang Z., Yang L., Sun S. (2023). Effect of Intestinal Microbiota Transplantation on Intestinal Flora and Inflammatory Factor Levels in Patients with Ulcerative Colitis. Infect. Drug Resist..

[10] Wangchuk P., Yeshi K., Loukas A. (2024). Ulcerative colitis: Clinical biomarkers, therapeutic targets, and emerging treatments. Trends Pharmacol. Sci..

[11] Jiang J., Ma Y., Zhou L., Han W., Liang Y., Dong J., Ding Y., Li W., Lei Q., Li J. (2025). Synergistic Genetic and Chemical Engineering of Probiotics for Enhanced Intestinal Microbiota Regulation and Ulcerative Colitis Treatment. Adv. Mater..

[12] Dilixiati Y., Aipire A., Song M., Nijat D., Wubuli A., Cao Q., Li J. (2024). The Potential Role of Plant Polysaccharides in Treatment of Ulcerative Colitis. Pharmaceutics.

[13] Guo Y., Li Y., Cao Q., Ye L., Wang J., Guo M. (2022). The Function of Natural Polysaccharides in the Treatment of Ulcerative Colitis. Front. Pharmacol..

[14] Zhang K., Zhu L., Zhong Y., Xu L., Lang C., Chen J., Yan F., Li J., Qiu J., Chen Y. (2023). Prodrug Integrated Envelope on Probiotics to Enhance Target Therapy for Ulcerative Colitis. Adv. Sci..

[15] Rong X., Shen C., Shu Q. (2024). Interplay between traditional Chinese medicine polysaccharides and gut microbiota: The elusive “polysaccharides-bond-bacteria-enzyme” equation. Phytother. Res..

[16] Son S.U., Kim T.E., Park J.H., Suh H.J., Shin K.S. (2024). Immunostimulating effects of ulvan type polysaccharide isolated from Korean Ulva pertusa in cyclophosphamide-induced immunosuppressed BALB/c mice. Int. J. Biol. Macromol..

[17] Song Q., Wang Y., Huang L., Xu L., Lang C., Chen J., Yan F., Li J., Qiu J., Chen Y. (2021). Review of the relationships among polysaccharides, gut microbiota, and human health. Food Res. Int..

[18] Hua Y., Liu R., Lu M., Guan X., Zhuang S., Tian Y., Zhang Z., Cui L. (2021). Juglone regulates gut microbiota and Th17/Treg balance in DSS-induced ulcerative colitis. Int. Immunopharmacol..

[19] Bao X., Tang Y., Lv Y., Fu S., Yang L., Chen Y., Zhou M., Zhu B., Ding Z., Zhou F. (2024). Tetrastigma hemsleyanum polysaccharide ameliorated ulcerative colitis by remodeling intestinal mucosal barrier function via regulating the SOCS1/JAK2/STAT3 pathway. Int. Immunopharmacol..

[20] Rong X., Zhu L., Shu Q. (2025). Synergistic gut microbiome-mediated degradation of *Astragalus membranaceus* polysaccharides and Codonopsis pilosula polysaccharides into butyric acid: A metatranscriptomic analysis. Microbiol. Spectr..

[21] Li M., Li S., Guo X., Guo C., Wang Y., Du Z., Zhang Z., Xie C., Ding K. (2021). Discrete genetic loci in human gut *Bacteroides* thetaiotaomicron confer pectin metabolism. Carbohydr. Polym..

[22] Kuhaudomlarp S., Pergolizzi G., Patron N.J., Henrissat B., Field R.A. (2019). Unraveling the subtleties of β-(1→3)-glucan phosphorylase specificity in the GH94, GH149, and GH161 glycoside hydrolase families. J. Biol. Chem..

[23] Schwalm N.D., Townsend G.E., Groisman E.A. (2017). Prioritization of polysaccharide utilization and control of regulator activation in *Bacteroides* thetaiotaomicron. Mol. Microbiol..

[24] Spragge F., Bakkeren E., Jahn M.T., Araujo E.B.N., Pearson C.F., Wang X., Pankhurst L., Cunrath O., Foster K.R. (2023). Microbiome diversity protects against pathogens by nutrient blocking. Science.

[25] Patnode M.L., Beller Z.W., Han N.D., Cheng J., Peters S.L., Terrapon N., Henrissat B., Le Gall S., Saulnier L., Hayashi D.K. (2019). Interspecies Competition Impacts Targeted Manipulation of Human Gut Bacteria by Fiber-Derived Glycans. Cell.

[26] Zhang D., Liu J., Cheng H., Wang H., Tan Y., Feng W., Peng C. (2022). Interactions between polysaccharides and gut microbiota: A metabolomic and microbial review. Food Res. Int..

[27] Sun G., Xiao Y., Yin H., Yu K., Wang Y., Wang Y. (2025). Oligosaccharide elicitors in plant immunity: Molecular mechanisms and disease resistance strategies. Plant Commun..

[28] Feng J., Qian Y., Zhou Z., Ertmer S., Vivas E.I., Lan F., Hamilton J.J., Rey F.E., Anantharaman K., Venturelli O.S. (2022). Polysaccharide utilization loci in *Bacteroides* determine population fitness and community-level interactions. Cell Host Microbe.

[29] Parada Venegas D., De la Fuente M.K., Landskron G., González M.J., Quera R., Dijkstra G., Harmsen H.J.M., Faber K.N., Hermoso M.A. (2019). Short Chain Fatty Acids (SCFAs)-Mediated Gut Epithelial and Immune Regulation and Its Relevance for Inflammatory Bowel Diseases. Front. Immunol..

[30] Zhang S., Zhou R., Xie X., Xiong S., Li L., Li Y. (2025). Polysaccharides from *Lycium barbarum*, yam, and sunflower ameliorate colitis in a structure and intrinsic flora-dependent manner. Carbohydr. Polym..

[31] Ozturk O., Celebi G., Duman U.G., Kupcuk E., Uyanik M., Sertoglu E. (2024). Short-chain fatty acid levels in stools of patients with inflammatory bowel disease are lower than those in healthy subjects. Eur. J. Gastroenterol. Hepatol..

[32] Mann E.R., Lam Y.K., Uhlig H.H. (2024). Short-chain fatty acids: Linking diet, the microbiome and immunity. Nat. Rev. Immunol..

[33] Peng W., He C.X., Li R.L., Qian D., Wang L.Y., Chen W.W., Zhang Q., Wu C.J. (2024). Zanthoxylum bungeanum amides ameliorates nonalcoholic fatty liver via regulating gut microbiota and activating AMPK/Nrf2 signaling. J. Ethnopharmacol..

[34] Zhang Z., Wang S., Zhang J., Zhang F., Wang H. (2025). Ultrasound-assisted non-starch polysaccharide extraction from bulbils of *Dioscorea opposita* maxim and its bioactivity on ulcerative colitis via improving the intestinal barrier and modifying the gut microbiota. Int. J. Biol. Macromol..

[35] Hu G., Shao T., Wang J., Zhang H., Shang X., Liu C., Wu J., Lv Q., Zhou Y., Chen L. (2025). Structural Characterization of a Branched Polysaccharide Isolated from *Thesium chinense* Turcz. and Its Ameliorative Effects on Ulcerative Colitis. J. Agric. Food Chem..

[36] Shao S., Wang D., Zheng W., Li X., Zhang H., Zhao D., Wang M. (2019). A unique polysaccharide from *Hericium erinaceus* mycelium ameliorates acetic acid-induced ulcerative colitis rats by modulating the composition of the gut microbiota, short chain fatty acids levels and GPR41/43 respectors. Int. Immunopharmacol..

[37] Huang M., Yu Q., Zhu Y., Zhang J., He Y., Zhu Y., Xiao X. (2025). Arabinoxylan from barley bran alleviates ulcerative colitis in mice through enhancement of mucosal barrier function and modulation of gut microbiota. Int. J. Biol. Macromol..

[38] Li Z., Saravanakumar K., Yao L., Kim Y., Choi S.Y., Yoo G., Lee P.J., Kim S., Cho N. (2025). Polysaccharide from *Asimina triloba* fruits alleviates DSS-induced ulcerative colitis by modulating inflammatory signaling and gut microbiota. NPJ Sci. Food.

[39] Liu T.Y., Han Z.W., Jia S.S., Ma K., Li M., Yi X., Zhu H., Fan J.H., Qiu H.W., Lv G.P. (2025). An acidic polysaccharide from *Lycium barbarum* L.: Isolation, purification, structural characterization, and therapeutic effects on ulcerative colitis. Int. J. Biol. Macromol..

[40] Yu B., Wang M., Teng B., Veeraperumal S., Cheung P.C., Zhong S., Cheong K.L. (2023). Partially Acid-Hydrolyzed Porphyran Improved Dextran Sulfate Sodium-Induced Acute Colitis by Modulation of Gut Microbiota and Enhancing the Mucosal Barrier. J. Agric. Food Chem..

[41] Zhang Y., Ji W., Qin H., Chen Z., Zhou Y., Zhou Z., Wang J., Wang K. (2025). Astragalus polysaccharides alleviate DSS-induced ulcerative colitis in mice by restoring SCFA production and regulating Th17/Treg cell homeostasis in a microbiota-dependent manner. Carbohydr. Polym..

[42] Zou M.Y., Wang Y.J., Liu Y., Xiong S.Q., Zhang L., Wang J.H. (2023). Huangshan Floral Mushroom Polysaccharide Ameliorates Dextran Sulfate Sodium-Induced Colitis in Mice by Modulating Th17/Treg Balance in a Gut Microbiota-Dependent Manner. Mol. Nutr. Food Res..

[43] Liu X., Zhang M., Chen S., Liu H., Ma H., Hu T., Luo P., Wei S. (2024). *Grifola frondosa* polysaccharide’s therapeutic potential in oxazolone-induced ulcerative colitis. Carbohydr. Polym..

[44] Peng G., Wang S., Zhang H., Xie F., Jiao L., Yuan Y., Ma C., Wu H., Meng Z. (2024). *Tremella aurantialba* polysaccharides alleviate ulcerative colitis in mice by improving intestinal barrier via modulating gut microbiota and inhibiting ferroptosis. Int. J. Biol. Macromol..

[45] Pan L., Ma M., Wang Y., Dai W., Fu T., Wang L., Shang Q., Yu G. (2024). Polyguluronate alleviates ulcerative colitis by targeting the gut commensal *Lactobacillus* murinus and its anti-inflammatory metabolites. Int. J. Biol. Macromol..

[46] Zhang J., Sun Z., Cheng L., Kang J., Liu Y., Zhao Y., Xiao M., Liu H., Zhu Q., Guo Q. (2025). Structural Characterization of Water-Soluble Pectin from the Fruit of *Diospyros lotus* L. and Its Protective Effects against DSS-Induced Colitis in Mice. J. Agric. Food Chem..

[47] Xiong F., Li H.Y., Yao H.L., Ou Y.H., Chan A.S.C., Wang S.P., Li H.J., Lan W.J. (2025). A galacturonic acid-rich polysaccharide from *Citrus medica* ‘fingered’ alleviated the dextran sulfate sodium-induced ulcerative colitis. Int. J. Biol. Macromol..

[48] Liu C., Hu B., Cheng Y., Guo Y., Yao W., Qian H. (2021). In-depth analysis of the mechanisms of aloe polysaccharides on mitigating subacute colitis in mice via microbiota informatics. Carbohydr. Polym..

[49] Lu H., Shen M., Chen Y., Yu Q., Chen T., Xie J. (2023). Alleviative effects of natural plant polysaccharides against DSS-induced ulcerative colitis via inhibiting inflammation and modulating gut microbiota. Food Res. Int..

[50] Jing Y., Wang Z., Cheng W., Fan H., Zheng K., Zheng Y., Wu L. (2025). Structure Characterization and Treatment Effect of *Zingiber officinale* Polysaccharide on Dextran Sulfate Sodium-Induced Ulcerative Colitis. Foods.

[51] Tan Y., Cao W., Yang L., Gong X., Li H. (2024). Structural characterization of the glucan from *Gastrodia elata* Blume and its ameliorative effect on DSS-induced colitis in mice. Int. J. Biol. Macromol..

[52] Qin L., Xu H., Cao J., Wang K., Zhang L., Yao M., Lin H., Qu C., Miao J. (2024). Alleviative effects of sulfated polysaccharide from *Ishige Okamurae* against DSS-induced ulcerative colitis via inhibiting inflammation and modulating gut microbiota. Int. J. Biol. Macromol..

[53] Wang J., Wang J., Kang Z., Pei J., Xia G., He R., Chen H. (2025). Structural elucidation of polysaccharide from fresh *Areca catechu* L. and its mitigation effect on DSS-induced colitis in mice. Int. J. Biol. Macromol..

[54] Feng Y., Chen S., Song Y., Liu S., Duan Y., Cai M., Kong T., Zhang H. (2024). A novel *Sagittaria sagittifolia* L. polysaccharides mitigate DSS-induced colitis via modulation of gut microbiota and MAPK/NF-κB signaling pathways. Int. J. Biol. Macromol..

[55] Sun X., Zhang T., Zhao Y., Yang H., Li Y., Sun X., Bian X. (2025). A novel glucomannan from *Bletilla striata* ameliorates colitis: Restores intestinal barrier, alleviates inflammation, and modulates the gut flora. Int. J. Biol. Macromol..

[56] Li Q., Wu W., Fang X., Chen H., Han Y., Liu R., Niu B., Gao H. (2022). Structural characterization of a polysaccharide from bamboo (*Phyllostachys edulis*) shoot and its prevention effect on colitis mouse. Food Chem..

[57] Xue H., Zha M., Li Z., Li L., Tan Y., Tan J. (2026). Recent advances in the ulcerative colitis effects of food and medicine homologous polysaccharides: Structural characterization, action mechanisms, and drug administration strategy. Int. J. Biol. Macromol..

[58] Fernandes P.A.R., Coimbra M.A. (2023). The antioxidant activity of polysaccharides: A structure-function relationship overview. Carbohydr. Polym..

[59] Bai G., Xie Y., Gao X., Xiao C., Yong T., Huang L., Cai M., Liu Y., Hu H., Chen S. (2024). Selective impact of three homogenous polysaccharides with different structural characteristics from *Grifola frondosa* on human gut microbial composition and the structure-activity relationship. Int. J. Biol. Macromol..

[60] Kim H.M., Song Y., Hyun G.H., Long N.P., Park J.H., Hsieh Y.S.Y., Kwon S.W. (2020). Characterization and Antioxidant Activity Determination of Neutral and Acidic Polysaccharides from *Panax Ginseng* C. A. Meyer. Molecules.

[61] Wardman J.F., Bains R.K., Rahfeld P., Withers S.G. (2022). Carbohydrate-active enzymes (CAZymes) in the gut microbiome. Nat. Rev. Microbiol..

[62] Wang W., Xu C., Liu Z., Gu L., Ma J., Hou J., Jiang Z. (2022). Physicochemical properties and bioactivity of polysaccharides from Isaria cicadae Miquel with different extraction processes: Effects on gut microbiota and immune response in mice. Food Funct..

[63] Ye J., Wang X., Wang K., Deng Y., Yang Y., Ali R., Chen F., Wu Z., Liao W., Mao L. (2020). A novel polysaccharide isolated from *Flammulina velutipes*, characterization, macrophage immunomodulatory activities and its impact on gut microbiota in rats. J. Anim. Physiol. Anim. Nutr..

[64] Pan L., Wang L., Zeng Z., Zhang Z., Zheng B., Zhang Y. (2025). Chemical structure and prebiotic activity of a *Dictyophora indusiata* polysaccharide fraction. Food Chem..

[65] Zhao Y., Wang Y., Ma Q., Wang D., Jiang Q., Wang P., Ge Z., Wang J., Qin P., Zhao X. (2024). Different microbiota modulation and metabolites generation of five dietary glycans during in vitro gut fermentation are determined by their monosaccharide profiles. Food Res. Int..

[66] Fu C., Ye K., Qiu Z., Ma G., Chen S., Xiao H. (2025). Structural Characterization and Gastrointestinal Fate of a High-Molecular-Weight *Hericium erinaceus* Polysaccharide during Digestion and Fermentation. J. Agric. Food Chem..

[67] Li J.H., Gu F.T., Yang Y., Zhao Z.C., Huang L.X., Zhu Y.Y., Chen S., Wu J.Y. (2024). Simulated human digestion and fermentation of a high-molecular weight polysaccharide from Lentinula edodes mushroom and protective effects on intestinal barrier. Carbohydr. Polym..

[68] Song W., Wang Y., Li G., Xue S., Zhang G., Dang Y., Wang H. (2023). Modulating the gut microbiota is involved in the effect of low-molecular-weight Glycyrrhiza polysaccharide on immune function. Gut Microbes.

[69] Cai Y., Si Z., Jiang Y., Ye M., Wang F., Yang X., Yu J., Gao X., Liu W. (2023). Structure-activity relationship of low molecular weight *Astragalus membranaceus* polysaccharides produced by *Bacteroides*. Carbohydr. Polym..

[70] Cao W., Liu Y., Chen N., Wang Y., Nushrat Y.M., Qiao S., Tang L., Sun Z., Lu R., Liu C. (2025). Preparation, characterization, fermentation properties of pectin with specific structures, and the analysis of microbial enzymes and genes involved in their degradation. Carbohydr. Polym..

[71] Ndeh D., Rogowski A., Cartmell A., Luis A.S., Baslé A., Gray J., Venditto I., Briggs J., Zhang X., Labourel A. (2017). Complex pectin metabolism by gut bacteria reveals novel catalytic functions. Nature.

[72] Huang W., Zhao M., Wang X., Tian Y., Wang C., Sun J., Wang Z., Gong G., Huang L. (2022). Revisiting the structure of arabinogalactan from *Lycium barbarum* and the impact of its side chain on anti-ageing activity. Carbohydr. Polym..

[73] Déjean G., Tauzin A.S., Bennett S.W., Creagh A.L., Brumer H. (2019). Adaptation of Syntenic Xyloglucan Utilization Loci of Human Gut Bacteroidetes to Polysaccharide Side Chain Diversity. Appl. Environ. Microbiol..

[74] Chen P., You Q., Li X., Chang Q., Zhang Y., Zheng B., Hu X., Zeng H. (2019). Polysaccharide fractions from Fortunella margarita affect proliferation of *Bifidobacterium* adolescentis ATCC 15703 and undergo structural changes following fermentation. Int. J. Biol. Macromol..

[75] Jadhav A., Jagtap S., Vyavahare S., Sharbidre A., Kunchiraman B. (2023). Reviewing the potential of probiotics, prebiotics and synbiotics: Advancements in treatment of ulcerative colitis. Front. Cell. Infect. Microbiol..

[76] Fang H., Fu L., Li X., Lu C., Su Y., Xiong K., Zhang L. (2021). Long-term efficacy and safety of monotherapy with a single fresh fecal microbiota transplant for recurrent active ulcerative colitis: A prospective randomized pilot study. Microb. Cell Fact..

[77] Meng X., Shu Q. (2024). Novel primers to identify a wider diversity of butyrate-producing bacteria. World J. Microbiol. Biotechnol..

[78] Fagundes R.R., Belt S.C., Bakker B.M., Dijkstra G., Harmsen H.J.M., Faber K.N. (2024). Beyond butyrate: Microbial fiber metabolism supporting colonic epithelial homeostasis. Trends Microbiol..

[79] Akiyama S., Nishijima S., Kojima Y., Kimura M., Ohsugi M., Ueki K., Mizokami M., Hattori M., Tsuchiya K., Uemura N. (2024). Multi-biome analysis identifies distinct gut microbial signatures and their crosstalk in ulcerative colitis and Crohn’s disease. Nat. Commun..

[80] Wang M., Fu R., Xu D., Chen Y., Yue S., Zhang S., Tang Y. (2024). Traditional Chinese Medicine: A promising strategy to regulate the imbalance of bacterial flora, impaired intestinal barrier and immune function attributed to ulcerative colitis through intestinal microecology. J. Ethnopharmacol..

[81] Singh V., Lee G., Son H., Koh H., Kim E.S., Unno T., Shin J.H. (2023). Butyrate producers, “The Sentinel of Gut”: Their intestinal significance with and beyond butyrate, and prospective use as microbial therapeutics. Front. Microbiol..

[82] Zhang K., Dong Y., Ding Y., Wang X., Liu T., Zhong W., Cao H. (2025). Illuminating prospects of probiotic *Akkermansia* muciniphila in intestinal inflammation and carcinogenesis. Microbiol. Res..

[83] Wen Y., Yang L., Wang Z., Liu X., Gao M., Zhang Y., Wang J., He P. (2023). Blocked conversion of *Lactobacillus* johnsonii derived acetate to butyrate mediates copper-induced epithelial barrier damage in a pig model. Microbiome.

[84] Czjzek M., Ficko-Blean E., Berrin J.G. (2023). A special issue of Essays in Biochemistry on current advances about CAZymes and their impact and key role in human health and environment. Essays Biochem..

[85] You Y., Gan B.K., Luo M., Zheng X., Dong N., Tian Y., Li C., Kong H., Gu Z., Yang D. (2025). Structure-Informed Insights into Catalytic Mechanism and Multidomain Collaboration in α-Agarase CmAga. J. Agric. Food Chem..

[86] Tang S., Wang T., Huang C., Lai C., Fan Y., Yong Q. (2020). Arabinogalactans from Larix principis-rupprechtii: An investigation into the structure-function contribution of side-chain structures. Carbohydr. Polym..

[87] Turroni F., Milani C., Duranti S., Mahony J., van Sinderen D., Ventura M. (2018). Glycan Utilization and Cross-Feeding Activities by Bifidobacteria. Trends Microbiol..

[88] Lin Q., Zhao W., Liang W., Wang X., Zeng J., Gao H., Li W. (2025). Ion type-based formation rules and functional properties of polysaccharide-starch aerogels with chitooligosaccharide, xanthan gum, and locust bean gum: A comparative study. Food Res. Int..

[89] Wang Y., Li C., Li J., Zhang S., Zhang Q., Duan J., Guo J. (2024). Abelmoschus manihot polysaccharide fortifies intestinal mucus barrier to alleviate intestinal inflammation by modulating *Akkermansia* muciniphila abundance. Acta Pharm. Sin. B.

[90] Song Q., Zhang K., Li S., Weng S. (2025). Trichosanthes kirilowii Maxim. Polysaccharide attenuates diabetes through the synergistic impact of lipid metabolism and modulating gut microbiota. Curr. Res. Food Sci..

[91] Bai Q., Zhao Z., Duan Y., Cai R., Chen Y., Zhou C., Tian X., Yang Y., Wu H., Li M. (2025). Enrichment of short-chain fatty acid-producing bacteria by pH-responsive sodium alginate and chitosan-encapsulated quercetin. Front. Microbiol..

[92] Liang L., Liu L., Zhou W., Yang C., Mai G., Li H., Chen Y. (2022). Gut microbiota-derived butyrate regulates gut mucus barrier repair by activating the macrophage/WNT/ERK signaling pathway. Clin. Sci..

[93] Carretta M.D., Quiroga J., López R., Hidalgo M.A., Burgos R.A. (2021). Participation of Short-Chain Fatty Acids and Their Receptors in Gut Inflammation and Colon Cancer. Front. Physiol..

[94] Koh A., De Vadder F., Kovatcheva-Datchary P., Bäckhed F. (2016). From Dietary Fiber to Host Physiology: Short-Chain Fatty Acids as Key Bacterial Metabolites. Cell.

[95] He X.D., Li M., Zuo X.D., Ni H.Y., Han Y.X., Hu Y.K., Yu J., Yang X.X. (2025). Kushenol I combats ulcerative colitis via intestinal barrier preservation and gut microbiota optimization. World J. Gastroenterol..

[96] Ungaro R., Mehandru S., Allen P.B., Peyrin-Biroulet L., Colombel J.F. (2017). Ulcerative colitis. Lancet.

[97] Peng K., Xiao S., Xia S., Li C., Yu H., Yu Q. (2024). Butyrate Inhibits the HDAC8/NF-κB Pathway to Enhance Slc26a3 Expression and Improve the Intestinal Epithelial Barrier to Relieve Colitis. J. Agric. Food Chem..

[98] Yao D., Dai W., Dong M., Dai C., Wu S. (2021). MUC2 and related bacterial factors: Therapeutic targets for ulcerative colitis. BioMedicine.

[99] Yang W., Xue Y., Zhu P., Jiang Z., He R., Tian H., Wang T., Xiao P., Lian W., Cao Q. (2025). Goblet cell-expressed microprotein FXYD3 determines gut homeostasis by maintaining mucus barrier integrity. Cell Rep..

[100] Krause F.F., Mangold K.I., Ruppert A.L., Leister H., Hellhund-Zingel A., Lopez Krol A., Pesek J., Watzer B., Winterberg S., Raifer H. (2024). *Clostridium* sporogenes-derived metabolites protect mice against colonic inflammation. Gut Microbes.

[101] Guo C., Guo D., Fang L., Sang T., Wu J., Guo C., Wang Y., Wang Y., Chen C., Chen J. (2021). Ganoderma lucidum polysaccharide modulates gut microbiota and immune cell function to inhibit inflammation and tumorigenesis in colon. Carbohydr. Polym..

[102] Liu M.-T., Zhang Y., Xiang C.G., Yang T., Wang X.H., Lu Q.K., Lu H.M., Fan C., Feng C.L., Yang X.Q. (2024). Methionine-choline deficient diet deteriorates DSS-induced murine colitis through disturbance of gut microbes and infiltration of macrophages. Acta Pharmacol. Sin..

[103] Shi J., Zhou J., Liu B., Lin K., Xie X., Han X., Sheng Y., Liu Y., He C., Zhou Y. (2024). Enzyme/ROS dual-sensitive nanoplatform with on-demand Celastrol release capacity for enhanced ulcerative colitis therapy by ROS scavenging, microbiota rebalancing, inflammation alleviating. J. Nanobiotechnol..

[104] Liu C., Hua H., Zhu H., Cheng Y., Guo Y., Yao W., Qian H. (2021). *Aloe* polysaccharides ameliorate acute colitis in mice via Nrf_2_/HO-1 signaling pathway and short-chain fatty acids metabolism. Int. J. Biol. Macromol..

[105] Bakky M.A.H., Tran N.T., Zhang M., Wang S., Zhang Y., Li S. (2025). Synergistic effects of butyrate-producing bacteria (*Clostridium senegalense* I5 or *Paraclostridium benzoelyticum* G5) and *Gracilaria lemaneiformis*-originated polysaccharides on the growth and immunity of rabbitfish. Int. J. Biol. Macromol..

