# Supplementary material for: The Structure–Decoding–Conversion–Effect Paradigm of Natural Polysaccharides for Gut Microbiota Remodeling in Ulcerative Colitis
Source: Nutrients. 2026 Apr 20;18(8):1297. doi: 10.3390/nu18081297 (PMC13119472; doi:10.3390/nu18081297)
Supplement: Supplementary file 1 [file nutrients-18-01297-s001.zip › nutrients-4220829-supplementary.pdf]

Table S1. Characteristics of intervention protocols for polysaccharides from different sources in animal models of ulcerative colitis.

Polysaccharide Source	Animal Model Establishment	Dosage Range	Administration Route	Intervention Duration & Timing
Dioscorea opposita Maxim [34]	C57BL/6J mice, administered 2.5% DSS in drinking water for 7 days, followed by normal water for 7 days. This cycle was repeated three times.	100, 200 mg/kg	Oral gavage	Administered from Day 1 of modeling, throughout the entire modeling and recovery periods.
Thesium chinense Turcz [35]	C57BL/6J mice, given free access to water containing 3% DSS. After successful modeling, mice were randomized into groups for intervention.	-	-	Intervention started after successful modeling (approx. Day 13) and continued until the end of the experiment.
Hericium erinaceus [36]	SD rats, fasted for 24h, anesthetized, and administered 1.5 ml of 4% acetic acid intra-anally, flushed after 15s.	0.6, 1.2 g/kg	Oral gavage	Intervention started on Day 2 post-modeling, lasting for 10 days.
Barley bran [37]	C57BL/6J mice, administered 2.5% DSS in drinking water for 7 days to induce colitis, switched to normal water from Day 8.	250 mg/kg	Oral gavage	Administered from Day 1 of modeling, continuing until the end of the experiment (total 11 days).
Asimina triloba [38]	C57BL/6J mice, administered 2.5% DSS in drinking water for 3 days.	100, 200, 400 mg/kg	Oral gavage	Administered after DSS exposure for 10 days.
Lycium barbarum L. [39]	C57BL/6J mice, administered 3.5% DSS in drinking water for 7 days (Day 1-7), then switched to sterile saline (Day 8-11).	200 mg/kg	Oral gavage	Administered from Day 1 for 11 days.
Porphyra haitanensis [40]	Kunming mice, administered 3% DSS in drinking water for 1 week (Day 8-14).	100, 200 mg/kg	Oral gavage	Intervention started after DSS cessation (Day 15-21), lasting 1 week.
Astragalus membranaceus [41]	BALB/c mice, administered 3% DSS in drinking water for 7 days (Day 8-14).	200, 400 mg/kg	Oral gavage	Administered starting 7 days before DSS induction (Day 1-14).
Floral Mushroom [42]	C57BL/6J mice, administered 3% DSS in drinking water for 7 days.	200, 400 mg/kg	Oral gavage	Administered from Day 1 of modeling for 7 days.
Grifola frondosa [43]	Kunming mice, sensitized with 3% OXZ-methanol on abdominal skin, followed by intra-intestinal injection of 1% OXZ-methanol.	80, 160, 320 mg/kg	Oral gavage	Administered daily for 7 consecutive days after successful modeling.
Tremella aurantialba [44]	C57BL/6 mice, administered 2.5% DSS in drinking water for 14 days, interspersed with cycles of 2 days DSS / 1 day normal water, repeated for 12 cycles.	20, 60 mg/kg	Oral gavage	Administered throughout the entire 12 cycles.

Laminaria japonica [45]	C57BL/6J mice, administered 2.0% DSS in drinking water in three repeated cycles, with DSS given for the first 5 days of each cycle.	100 mg/kg	Oral gavage	Administered daily throughout the entire experimental period (32 days).
Diospyros lotus L. [46]	C57BL/6 mice, administered 2.5% DSS in drinking water for 7 days.	100, 300 mg/kg	Oral gavage	Administered from Day 1 of modeling for 7 days.
Citrus medica [47]	C57BL/6 mice, given free access to water containing 2.5% DSS for 9 days.	50, 100, 200 mg/kg	Oral gavage	Administered concurrently with DSS induction for 9 days.
Aloe [48]	C57BL/6 mice, administered 2.5% DSS in drinking water from Day 15 to Day 21.	50, 100 mg/kg	Oral gavage	Administered from Day 1 of the experiment for 21 days.
Cyclocarya paliurus [49]	C57BL/6 mice, administered 3% DSS in drinking water for 7 days (Day 7-14).	200 mg/kg	Oral gavage	Administered from Day 1 of the experiment for 14 days.
Dioscorea opposita Maxim [49]	C57BL/6 mice, administered 3% DSS in drinking water for 7 days (Day 7-14).	200 mg/kg	Oral gavage	Administered from Day 1 of the experiment for 14 days.
Zingiber officinale Roscoe [50]	Kunming mice, given free access to water containing 2.5% DSS for 9 days.	500, 1000 mg/kg	Oral gavage	Administered concurrently with DSS induction for 9 days.
Gastrodia elata Blume [51]	C57BL/6J mice, administered 3% DSS in drinking water for 5 days, followed by normal water for 3 days.	200 mg/kg	Oral gavage	Administered from Day 1 of modeling for 8 days.
Ishige okamurae [52]	C57BL/6J mice, administered 3% DSS in drinking water for 5 days.	200, 600 mg/kg	Oral gavage	Administered starting 7 days before DSS induction, lasting 12 days.
Areca catechu L. [53]	C57BL/6J mice, administered DSS in drinking water for 7 days.	100, 200 mg/kg	Oral gavage	Administered from Day 1 of modeling for 7 days.
Sagittaria sagittifolia L. [54]	BALB/c mice, administered 5% DSS in drinking water for 7 days (Day 1-7), followed by normal water (Day 8-15).	100, 200, 400 mg/kg	Oral gavage	Administered from Day 8 for 8 days.
Bletilla striata [55]	Kunming mice, administered 3% DSS in drinking water for 15 days (Day 8-22).	100, 200 mg/kg	Oral gavage	Administered starting 7 days before DSS induction (Day 1-22).
Phyllostachys edulis [56]	C57BL/6 mice, administered 4% DSS in drinking water for 7 days.	100, 200, 400 mg/kg	Oral gavage	Administered starting 7 days before DSS induction, throughout the modeling period.

Notes: DSS, dextran sulfate sodium; OXZ, oxazolone; SD rats, Sprague-Dawley rats; "-" indicates not specified in the original reference. Interventions were administered via oral gavage unless otherwise stated. The timing of intervention is expressed relative to disease modeling.
